# An mRNA mix redirects dendritic cells towards an antiviral program, inducing anticancer cytotoxic stem cell and central memory CD8^+^ T cells

**DOI:** 10.3389/fimmu.2023.1111523

**Published:** 2023-02-13

**Authors:** Wout de Mey, Hanne Locy, Kirsten De Ridder, Phaedra De Schrijver, Dorien Autaers, Asma Lakdimi, Arthur Esprit, Lorenzo Franceschini, Kris Thielemans, Magali Verdonck, Karine Breckpot

**Affiliations:** Laboratory for Molecular and Cellular Therapy, Department of Biomedical Sciences, Vrije Universiteit Brussel, Brussels, Belgium

**Keywords:** cancer, CD40 ligand, dendritic cell, interferon-gamma, interleukin-10, interleukin-12, mRNA, toll-like receptor 4

## Abstract

Dendritic cell (DC)-maturation stimuli determine the potency of these antigen-presenting cells and, therefore, the quality of the T-cell response. Here we describe that the maturation of DCs *via* TriMix mRNA, encoding CD40 ligand, a constitutively active variant of toll-like receptor 4 and the co-stimulatory molecule CD70, enables an antibacterial transcriptional program. Besides, we further show that the DCs are redirected into an antiviral transcriptional program when CD70 mRNA in TriMix is replaced with mRNA encoding interferon-gamma and a decoy interleukin-10 receptor alpha, forming a four-component mixture referred to as TetraMix mRNA. The resulting TetraMixDCs show a high potential to induce tumor antigen-specific T cells within bulk CD8^+^ T cells. Tumor-specific antigens (TSAs) are emerging and attractive targets for cancer immunotherapy. As T-cell receptors recognizing TSAs are predominantly present on naive CD8^+^ T cells (T_N_), we further addressed the activation of tumor antigen-specific T cells when CD8^+^ T_N_ cells are stimulated by TriMixDCs or TetraMixDCs. In both conditions, the stimulation resulted in a shift from CD8^+^ T_N_ cells into tumor antigen-specific stem cell-like memory, effector memory and central memory T cells with cytotoxic capacity. These findings suggest that TetraMix mRNA, and the antiviral maturation program it induces in DCs, triggers an antitumor immune reaction in cancer patients.

## Introduction

Cancer-specific CD8^+^ cytotoxic T cells are key in rejecting cancer cells (1). Therefore, studies focusing on vaccination strategies to activate CD8^+^ T cells are crucial (2). Herein, mature dendritic cells (DCs) play a central role, as these innate immune cells can present tumor antigen-derived peptides in major histocompatibility complex (MHC) proteins to the T-cell receptor (TCR) and can provide co-stimulation for T-cell activation (3).

It is well-established that the stimuli used for DC-maturation determine the immune stimulatory capacity of the antigen-presenting DCs and therefore the strength and quality of the T-cell response (4). Tumor-specific antigens (TSAs) are emerging as attractive targets in cancer immunotherapy (5). These TSAs are considered non-self and trigger high avidity tumor-specific T-cell responses, predominantly found in naive CD8^+^ T cells (6, 7). A high level of peptide-MHC (pMHC)-complexes and optimal co-stimulation are required to prime naive CD8^+^ T cells (T_N_) (8). Increasing evidence shows that to ensure persistent anticancer immunity, a shift of CD8^+^ T_N_ cells to stem cell-like memory T cells (T_SCM_) and central memory T cells (T_CM_) is required (9–11). This highlights the need to deeply investigate the DC-maturation protocol.

A wide range of stimuli has been explored to mature DCs *ex vivo* or *in vivo* providing a wealth of information indicating which DC features are required to induce anticancer immunity (10). Compelling results showed that toll-like receptor 4 (TLR4) combined with CD40 signaling on DCs stimulates the expression of crucial CD8^+^ T-cell activating proteins, among which CD70 and interleukin (IL)-12 (12–14). The co-stimulatory ligand CD70 binds the receptor CD27 on T cells and stimulates effector and memory T-cell differentiation of CD8^+^ T cells (15). Moreover, CD27/CD70 interaction reverses tolerance to self-antigens (16). Also, IL-12 enhances the activation of CD8^+^ T cells (17). It stimulates the presentation of antigens by DCs to CD8^+^ T cells, thereby avoiding anergy (18). This cytokine further promotes CD8^+^ T-cell proliferation, effector functions, among which the production of cytokines like interferon-gamma (IFN-γ) and cytolytic factors like perforin and granzyme B (19–22). Production of IFN-γ in response to IL-12, in turn, acts on DCs, stimulating IL-12 secretion and as such provides a positive feedback loop (23). For that reason, IFN-γ has been introduced in several DC-maturation protocols, showing its effects on IL-12 production in combination with cytokine, CD40 or TLR-initiated signals (24–26). Notably, IL-10 is also produced by DCs upon maturation by CD40 and TLR(4) signaling (27). This cytokine stimulates immune tolerance in an autocrine way by inhibiting the production of pro-inflammatory cytokines (*e.g.*, IL-1β, IL-6, IL-12 and IFN-β) while stimulating the production of anti-inflammatory cytokines (*e.g*., IL-10) (28, 29). Therefore, IL-10 is to be avoided during the T-cell activation, as evidenced by the positive effects of blocking IL-10 during cancer vaccination (30, 31).

mRNA has proven to be a versatile platform to engineer DCs *ex vivo* or *in vivo* for T-cell activation (32, 33). From 1995 onwards, many research groups have shown that mRNA encoding tumor-associated antigens (TAAs) or TSAs can be used to activate anticancer immunity (34–37). Moreover, mRNA encoding cytokines, co-stimulatory molecules and inhibitory receptor blocking moieties has been used to increase the DC’s T-cell activating capacity, enhancing the magnitude, breadth and/or durability of anticancer immune responses (38–41). In clinical practice, monocyte-derived DCs (moDCs) electroporated with mRNA encoding TAAs in conjunction with TriMix mRNA, a mix of three mRNA molecules encoding CD40 ligand (CD40L), CD70, and a constitutively active variant of TLR4 (caTLR4), has shown an unprecedented capacity to increase the life expectancy of stage III/IV melanoma patients. Either when used as a monotherapy or in combination with blockade of cytotoxic T lymphocyte-associated antigen-4 (CTLA-4), an inhibitory receptor that hampers anticancer immune activity (42, 43). Using these TriMix mRNA modified moDC, we have documented that the secretion of IL-12 by the cellular therapy is most important. We observed that the capacity of TriMix-DC to secrete IL-12p70 correlated with the detection of vaccine-antigen specific CD8^+^ T cells at the vaccine-induced DTH site post vaccination. IL-12p70 is known to be a pivotal cytokine for numerous immunological processes pertinent to vaccine-based antitumor activity (44). These and other studies supported that IL-12 is a hallmark of an effective moDC-vaccine, capable of stimulating T cells (44–46).

In the present study, we investigated whether TetraMix mRNA, a four-component mRNA mixture, including CD40L, caTLR4, IFN-γ and decoy interleukin-10 receptor alpha (dIL10Rα), could enhance IL-12 production in electroporated moDCs. The four components of TetraMix mRNA have been selected due to their ability to induce activation and maturation in moDCs. In particular, CD40L will induce DC-activation through the ligation of the endogenous CD40, leading to upregulation of co-stimulatory molecules and enhancing cytokine production. Expression of caTRL4 will lead to DC maturation and improve their ability to stimulate antigen-specific T cells. IFN-γ mRNA plays an important role in the transcriptional activation of IL-12p70, which will enhance the activation of CD8^+^ T cells. The last component of TetraMix mRNA is dI10Rα mRNA. IL-10 produced by DCs can influence the DC-maturation process and down-regulate IL-12 production. IL-10 binds first to the IL10Rα protein before forming a complete and functional hetero-tetrameric signaling complex. The engineered dIL10Rα variant that is used in the TetraMix lacks the intracellular signal transduction domain. The dIL10Rα protein competes with the endogenous IL10Rα chain for binding to IL-10 and therefore behaves as an IL-10 trap. Overexpression of the dIL10Rα variant, binds to IL-10 but fails to transmit any further inhibitory signal to the DC.

In this work, moDCs electroporated with TetraMix mRNA, were thoroughly examined in terms of transcriptional profile, phenotype and capacity to produce IL-10 and IL-12, as well as capacity to activate tumor antigen-specific CD8^+^ cytotoxic T cells. moDCs electroporated with TetraMix mRNA (TetraMixDCs) were benchmarked to moDCs electroporated with TriMix mRNA (TriMixDCs). Our results show induction of antibacterial versus antiviral transcriptional program when using TriMix versus TetraMix mRNA for DC-maturation. Furthermore, antigen presenting TetraMixDCs show increased expression of various T-cell stimulatory proteins compared to TriMixDCs and were effective in activating tumor antigen-specific CD8^+^ cytotoxic T cells. These findings suggest that TetraMix mRNA, and the antiviral maturation program it induces, has a high potential for DC-based immunotherapy applications.

## Results

### Antibacterial versus antiviral transcriptional program initiation via respectively either TriMix or TetraMix mRNA in dendritic cell

**Figure 1:**
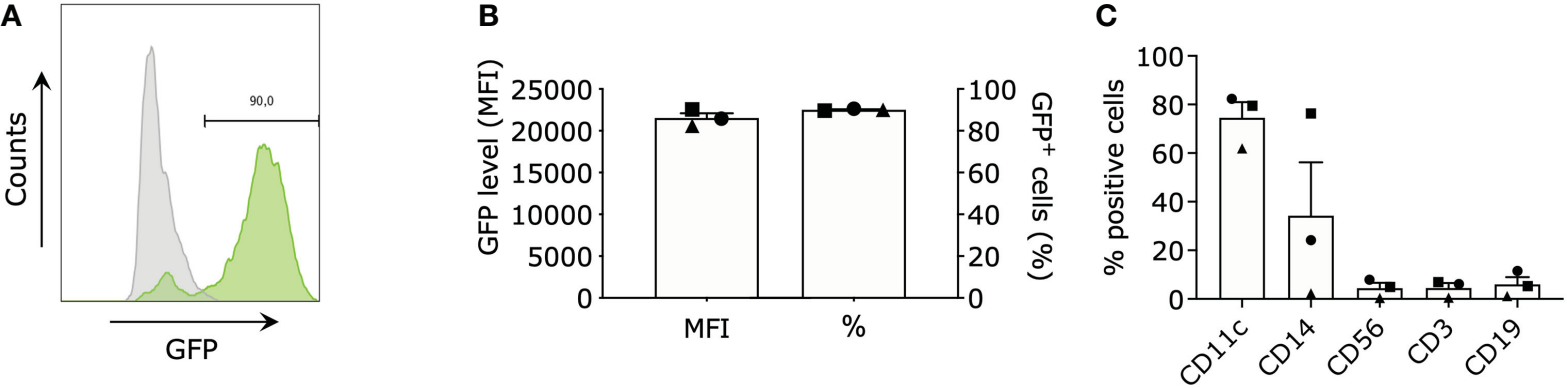
Purity and electroporation efficiency of monocyte-derived dendritic cells (moDCs). moDCs were electroporated with green fluorescent protein (GFP) mRNA (control), TriMix mRNA, or TetraMix mRNA to evaluate the reprogrammable capacities of TetraMix mRNA. **(A)** Histogram showing the shift of GFP^+^ cells (green) after electroporation (representative data). **(B)** The two bar graphs illustrate the expression of GFP in moDCs electroporated with GFP mRNA, for three different donors. More specifically, the graphs represent the percentage of GFP-positive cells (right bar) and the geometric mean fluorescence intensity of GFP (left bar), 24 hours post-electroporation. **(C)** The bar graph shows the expression of the cell surface markers: CD11c, CD14, CD56, CD3 and CD19 on the moDCs of three different donors, (percentage of positive cells). The bar graphs in **(B, C)** show the mean value ± standard error of the mean for three independent experiments (n=3). Each symbol represents the data of an individual donor.

We performed targeted gene expression profiling on moDCs electroporated with mRNA encoding green fluorescent protein (GFP), TriMix mRNA (CD40L, caTLR4 & CD70) and TetraMix mRNA (CD40L, caTLR4, IFN-γ & dIL10Rα) using the Nanostring platform. In all conditions, we electroporated 4x10^6^ moDCs with an equal amount of total mRNA (concentration in electroporation medium: 100 μg/ml). We initiated the transcriptome analysis 24 hours after electroporation, as translation of the delivered mRNA has taken place at that time, as evidenced by expression of GFP in moDCs electroporated with GFP mRNA (**Figures 1A, B**). We first assessed the purity of moDCs before electroporation and before transcriptome analysis using flow cytometry, evaluating the expression of CD11c, CD14 (monocytes), CD3 (T cells), CD19 (B cells) and CD56 (NK cells) on the cells (**Figure 1C**).

**Figure 2:**
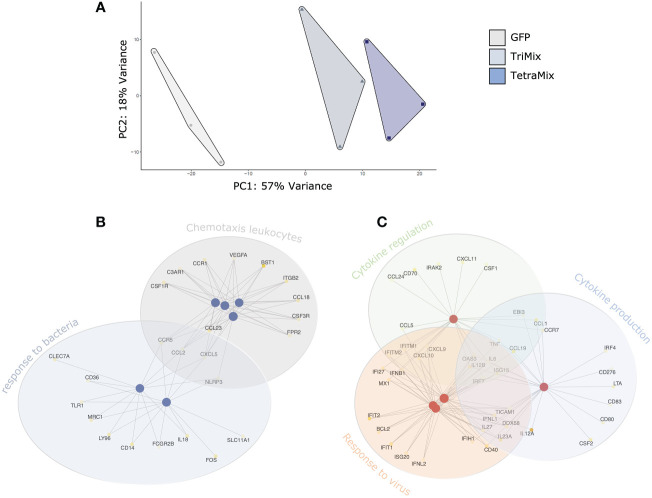
TetraMix mRNA induces an antiviral transcriptional program. Gene ontology network plots reveal the transcriptional alterations in biological processes between TriMix- and TetraMix-reprogrammed DCs (referred to as ‘TriMixDCs’ and ‘TetraMixDCs’). **(A)** Principal component analysis (PCA) of the transcriptomic analysis on dendritic cells 24 hours after electroporation (for each condition, *i.e.* GFP, TriMix and TetraMix, n=3). **(B)** Network plot of the top 6 biological processes and the involved genes that are downregulated in TetraMixDCs compared to TriMixDCs. Each node represents a gene set (*i.e*., a GO term). **(C)** Network plot of the top 5 biological processes and the involved genes that are upregulated in TetraMixDCs compared to TriMix DCs. Each node represents a gene set (*i.e.*, a GO term). Statistical significance is quantified using the gene score (-log_10_ adjusted p-value) and visualized *via* a yellow-to-white color palette, ranging from highest to lowest significance respectively.

A Principal Component Analysis (PCA) was performed for exploration of the targeted transcriptome data. The first principal component explains 57% of the overall variance, clearly separating the delivery of GFP, TriMix and TetraMix mRNA to moDCs. This PCA thus indicates that the delivery of the different mRNA’s induces distinct transcriptional profiles (**Figure 2A**). Next, we performed gene ontology analysis to gain a broader understanding of the transcriptional program initiated in moDCs by TriMix versus TetraMix mRNA. We observed that changes induced by TriMix mRNA could be defined as a response to bacteria, including changes in the expression of genes involved in sensing lipopolysaccharide (LPS) and the attraction of leukocytes (**Figure 2B**). In contrast, we observed that the changes initiated by TetraMix mRNA in moDCs were characteristic of viral defense response, as evidenced by the upregulation of many IFN-stimulated genes (ISGs) coinciding with changes in the expression of genes involved in cytokine regulation and production (**Figure 2C**).

### TetraMix mRNA engineered dendritic cells express genes characteristic of immunogenic antigen-presenting cells

**Figure 3:**
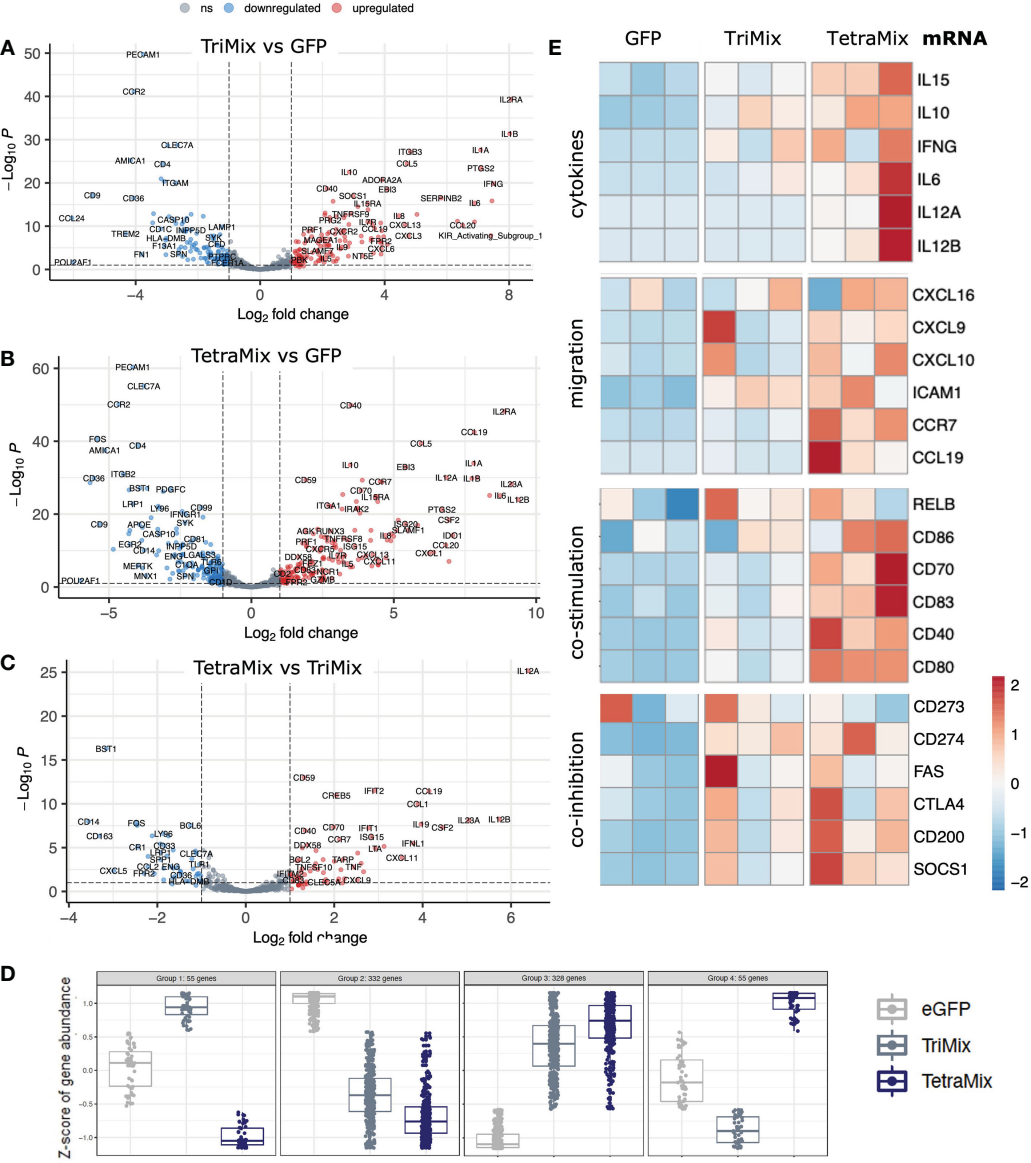
TetraMix mRNA reprogrammed DCs express genes characteristic of immunogenic antigen-presenting cells. a-c) Volcano plots displaying differential gene expression restricted to an adjusted p-value <0.1 and a log_2_ fold change >2 or <-2 calculated *via* DESeq2 in R. **(A)** Differential expression of genes measured in TriMixDCs compared to the control (GFP) DCs. **(B)** Differential expression of genes measured in TetraMixDCs compared to the control (GFP) DCs. **(C)** Differential gene expression of TetraMixDCs compared to TriMixDCs. **(D)** Expression pattern analysis depicted in dot plots separating genes into four categories based on similarities in transcriptional changes between all three conditions. **(E)** Heatmaps representative of gene expression between GFP, TriMixDCs and TetraMixDCs. Gene clusters were selected based on the literature and are considered to be representative of an immunogenic antigen-presenting cell signature. These clusters include cytokines, migration, co-stimulation and co-inhibition encoding genes.

To analyze the transcriptome in more detail, differential expression of genes between moDCs electroporated with either TriMix or TetraMix mRNA and moDCs electroporated with GFP was analyzed and visualized in volcano plots. A total of 258 genes were differentially expressed between TriMixDCs and moDCs electroporated with GFP (**Figure 3A**; **Table S1**). TetraMixDCs showed 343 genes that were differentially expressed compared to moDCs electroporated with GFP (**Figure 3B**; **Table S2**). Moreover, a volcano plot was generated to scrutinize transcriptomic changes between TriMixDCs and TetraMixDCs indicating 105 differentially expressed genes (**Figure 3C**; **Table S3**). When observing the transcriptional patterns between all three conditions we found that 328 and 332 genes were up- and downregulated, respectively, in TriMixDCs and TetraMixDCs compared to moDCs electroporated with GFP mRNA. A total of 110 genes showed an opposite gene expression shift between the TriMixDCs and TetraMixDCs compared to moDCs modified to express GFP (**Figure 3D**).

Based on literature, we selected four gene groups that could be considered determinants of the immunogenicity of DCs. These were plotted in heatmaps to compare GFP, TriMix or TetraMix mRNA electroporated moDCs (**Figure 3E**). The selected genes encode cytokines (IL-6, IL-10, IL-12, IL-15 and IFN-γ), proteins involved in chemotaxis (CXCL9, CXCL10, CXCL16, CCL19, CCR7 and ICAM1), in co-stimulation (RELB, CD40, CD70, CD80, CD83 and CD86) or in co-inhibition (CD273 [PD-L2], CD274 [PD-L1], CD95 [FAS], CD154 [CTLA-4], CD200 [OX-2] and SOCS1) (11, 47). It illustrates that TetraMixDCs showed increased expression of genes encoding cytokines and proteins driving cell migration and T-cell co-stimulation compared to TriMixDCs (**Figure 3E**). We further observed that genes encoding proteins that could hamper T-cell activation were increased in TetraMixDCs, though the difference in expression compared to TriMixDCs was less evident (**Figure 3E**).

### TetraMix engineered dendritic cells phenotype is enriched with high protein expression of co-stimulatory proteins

The phenotype of TriMixDCs or TetraMixDCs was evaluated to validate the findings of the transcriptome analysis, comparing to the phenotype of moDCs electroporated with mRNA encoding a truncated variant of nerve growth factor receptor (ControlDCs), which serves as a negative control.

**Figure 4:**
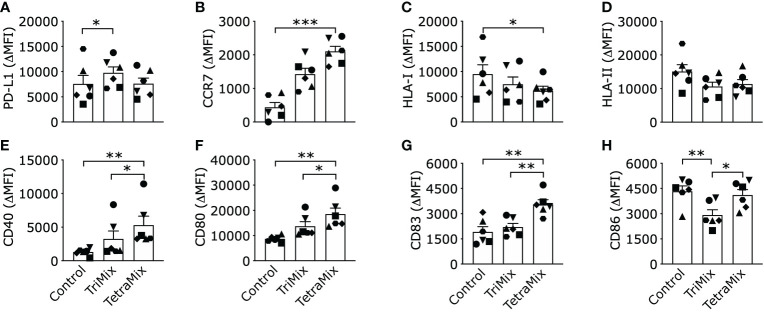
Phenotype of dendritic cells reprogrammed with TriMix or TetraMix mRNA. The bar graphs show the expression of PD-L1 **(A)**, CCR7 **(B)**, HLA-I **(C)**, HLA-II **(D)**, CD40 **(E)**, CD80 **(F)**, CD83 **(G)** and CD86 **(H)**, for three conditions, namely, tNGFR electroporated moDCs (Control), moDCs electroporated with TriMix or TetraMix mRNA. Results are expressed as the mean value of the delta-MFI ± standard error of the mean for six independent experiments (n=6). The delta-MFI is calculated from the MFI of the cells expressing the marker of interest subtracted by the MFI of the cells stained with the isotype control. Each symbol represents the data of an individual donor. Statistical significance is indicated as follows: *p < 0.05, **p < 0.01, ***p < 0.001, by Friedman’s test.

We observed that expression of the inhibitory ligand PD-L1 was significantly enhanced compared to ControlDCs on TriMixDCs yet not TetraMixDCs (**Figure 4A**). Furthermore, we observed that the C-C chemokine receptor 7 (CCR7), which is key for the migration of DCs to the T-cell zone of lymphoid organs, was significantly upregulated only on TetraMixDCs (**Figure 4B**). We observed a decreased expression of human leukocyte antigen (HLA) class I protein, which reached significance for HLA-I in TetraMixDCs (**Figure 4C**). The targeted transcriptome analysis showed no significant difference for HLA-I genes, such as HLA-A, HLA-B and HLA-C. We observed a downregulation from HLA class II proteins (HLA -DPA1, -DPB1, -DRB3, -DRB4, -DMA, -DMB, -DRA) in transcriptome analysis (**Tables S1**, **S2**), but no significant difference from moDC phenotype screening (**Figure 4D**). Confirming that the transcriptome analysis, in which we observed that expression of co-stimulatory proteins such as CD40, CD80 and CD83 on the surface of TetraMixDCs significantly increased compared to both ControlDCs and TriMixDCs (**Figures 4E–G**). This finding was not extended to the expression of CD86. We observed a significant decrease in CD86 expression on TriMixDCs. As a result, CD86 expression was significantly different compared to TetraMixDCs mRNA, which showed similarly high levels of CD86 expression as ControlDCs (**Figure 4H**).

### Dendritic cells reprogrammed with TetraMix mRNA produce high levels of IL-12

To support our claim that TetraMixDCs induce production of IL-12 and to validate the findings of the transcriptomic changes, we measured the levels of IL-12p70 and IL-10 secretion in the supernatants of moDCs, both between 0-24h and 24-48h post-electroporation with TriMix or TetraMix mRNA.

**Figure 5:**
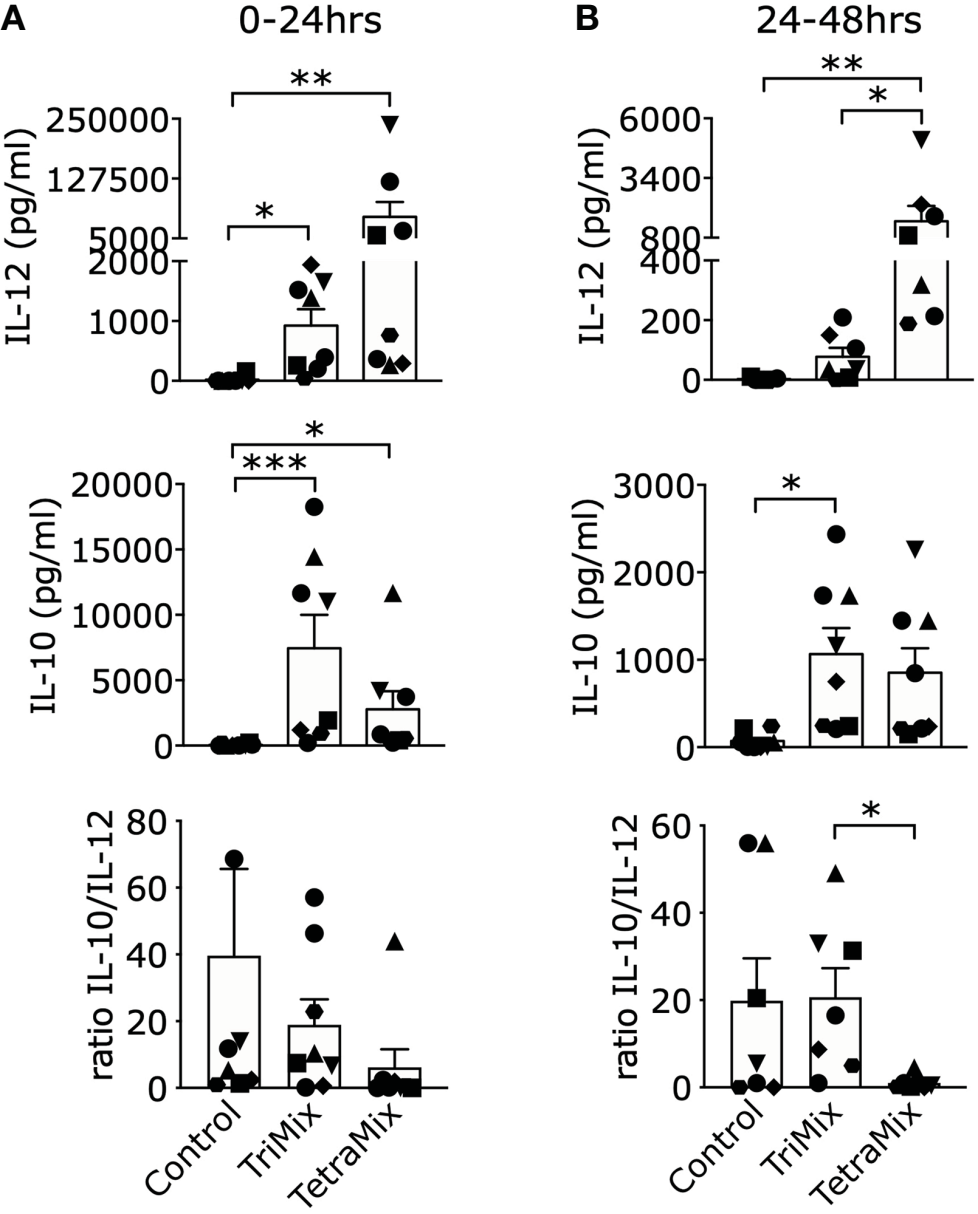
Interleukin-12 (IL-12) and IL-10 cytokine secretion profiles of DCs reprogrammed with TriMix or TetraMix mRNA. **(A)** Bar graphs representing the concentration of IL-12 or IL-10 detected in the supernatants of moDCs electroporated with tNGFR (control mRNA), TriMix or TetraMix mRNA, 24 hours post-electroporation. The lower graph shows the IL-10/IL-12 concentration ratio. **(B)** Bar graphs representing the concentration of IL-12 or IL-10 detected in the supernatants of control DCs, TriMixDCs or TetraMixDCs between 24 and 48 hours post-electroporation. The lower graph shows the IL-10/IL-12 concentration ratio. Results are shown as mean value ± standard error of the mean and for 8 independent experiments (n=8). Each symbol represents the data of an individual donor. Statistical significance is indicated as follows: *p < 0.05, **p < 0.01, ***p < 0.001, by Friedman’s test.

We expected a high amount of secreted IL-12p70 as IL-12p35 (IL12A) is in the top ten and IL12p40 (IL12B) in the top 25 upregulated genes after comparing moDCs electroporated with GFP or TetraMix mRNA (**Table S2**). IL12A was the most significant upregulated gene by comparing TetraMixDCs and TriMixDCs whereas IL12B is in the top ten (**Figure 3C**; **Table S3**). We observed a significant increase of IL-12p70 secretion by TetraMixDCs at both time points compared to the moDCs electroporated with control mRNA (**Figure 5A**). Between 24h and 48h, a higher IL-12 secretion level was observed for TetraMixDCs compared to TriMix-DCs (**Figure 5B**), validating the differential gene expression.

Based on the transcriptome data, we expect a high secretion of IL-10 by TriMixDCs and TetraMixDCs as it can be found in the top ten of the most upregulated genes (**Tables S1**, **S2**). Indeed, both TriMixDCs and TetraMixDCs show significant secretion of IL-10 after 24h and between 24h and 48h compared with the moDCs electroporated with control mRNA (**Figures 5A, B**). Next, we compared the IL-10/IL-12 ratio; showing that this ratio is significantly lower between 24h and 48h for TetraMixDCs compared to TriMixDCs.

### Dendritic cells matured with TetraMix mRNA and presenting Melan-A/MART-1 (A27L) expand antigen-specific CD8^+^ T cells

**Figure 6:**
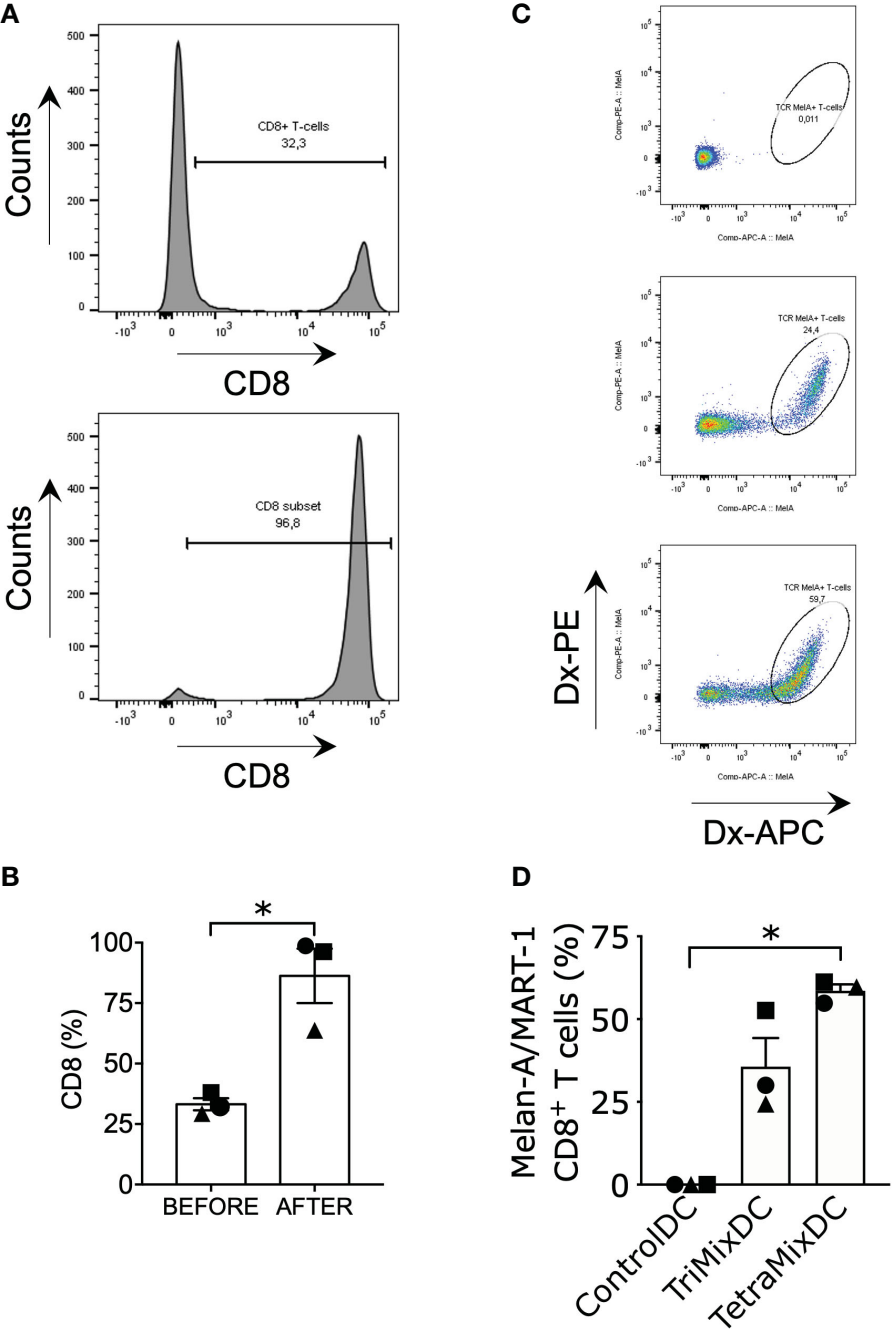
TetraMixDCs generate Melan-A/MART-1-specific T cells from bulk CD8^+^ autologous T cells. Dendritic cells co-electroporated with TriMix mRNA or TetraMix mRNA and Melan-A/Mart-1 mRNA were co-cultured with sorted autologous CD8^+^ T cells. DCs electroporated with tNGFR were used as negative control. **(A)** Histograms showing the shift in CD8^+^ T cell percentage prior (upper graph) and post (lower graph) CD8^+^ T cell isolation (representative data). **(B)** The bar graph represents the purity of CD8^+^ T cells prior (left bar) and after (right bar) CD8^+^ T cell isolation. **(C)** Flow cytometry gating strategy for double positive dextramer staining of Melan-A/MART-1-specific CD8^+^ T cells. **(D)** The bar graph represents the percentage of Melan-A/MART-1-specific CD8^+^ T cells at the end of the stimulation assay (21 days, weekly stimulation, total of 3 stimulation rounds). Results are shown as mean value ± standard error of the mean for 3 independent experiments (n=3). Each symbol represents the data of an individual donor. Statistical significance is indicated as follows: *p < 0.05, by Friedman’s test.

As TriMixDCs and TetraMixDCs showed mature phenotypes and activated cytokine production, we investigated whether these moDCs could induce tumor antigen-specific CD8^+^ T cells. Therefore, moDCs from HLA-A2^+^ healthy donors were co-electroporated with Melan-A/MART-1 (A27L) mRNA and either control mRNA (tNGFR) or, TetraMix and TriMix mRNA. Co-cultures were set up with autologous purified CD8^+^ T cells at a moDC to T cell ratio of 1 to 10 and re-stimulation was performed twice. The purity of CD8^+^ T cells after cell sorting was assessed by flow cytometry (**Figures 6A, B**). After 3 stimulation rounds, the percentage of dextramer positive, Melan-A/MART-1 (A27L)-specific CD8^+^ T cells were determined (**Figure 6C**). We observed that TetraMixDCs were the most potent in activating Melan-A/MART-1 (A27L)-specific CD8^+^ T cells compared to TriMixDCs and moDCs electroporated with control mRNA (**Figure 6D**).

### Melan-A/MART-1 (A27L)-specific CD8^+^ T cells expanded from a naive population by TetraMixDC acquire a stem cell-like, effector and central memory phenotype

**Figure 7:**
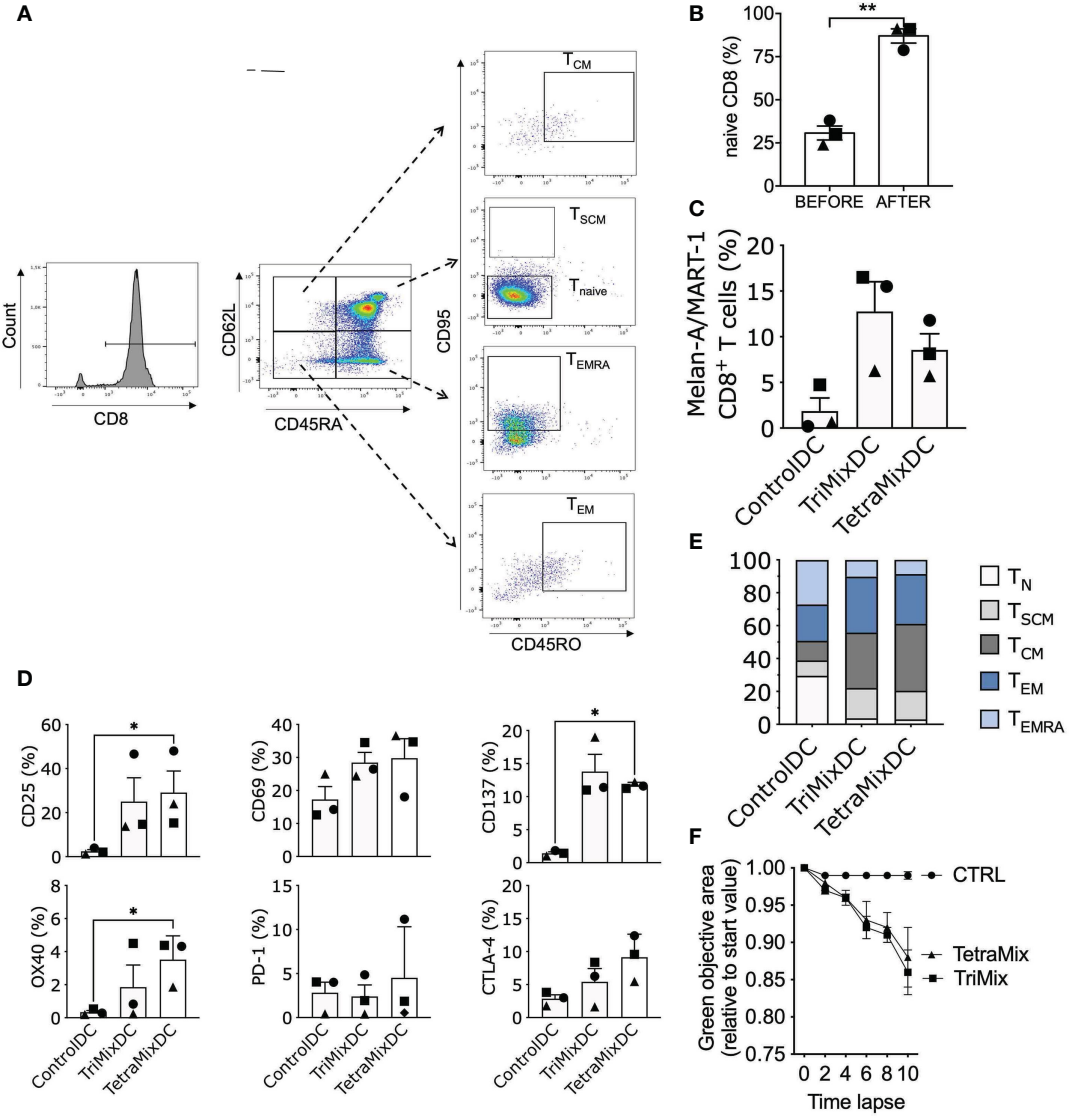
TetraMixDC stimulated naive T cells acquire an activated memory-like phenotype capable of inducing tumor cell death. DC co-electroporated with Melan-A/MART-1 mRNA and tNGFR (control), TriMix or TetraMix mRNA were co-cultured with sorted autologous naive CD8^+^ T cells (CD45RA^+^CD62L^+^CD45RO^-^CD95^-^). **(A)** Flow cytometry gating strategy for naive T cells (CD45RA^+^CD62L^+^CD45RO^-^CD95^-^), Stem cell-like memory (SCM) T cells (CD45RA^+^CD45RO^-^CD62L^+^CD95^+^), Central memory (CM) T cells (CD45RA^-^CD45RO^+^CD62L^+^CD95^+^), Effector memory (EM) T cells (CD45RA^-^CD45RO^+^CD62L^-^CD95^+^) and Effector memory re-expressing CD45RA (EMRA) T cells (CD45RA^+^CD45RO^-^CD62L^-^CD95^+^). **(B)** Bar graph showing the purity of naive CD8^+^ T cells: percentage of naive CD8^+^ T cell prior (left bar) and post (right bar) cell isolation. **(C)** Bar graph representing the percentage of Melan-A/MART-1-specific CD8^+^ T cells at the end of the stimulation assay (21 days, weekly stimulation, total of 3 stimulation rounds). d) Bar graphs representing the percentage of activation markers on CD8^+^ T cells after the stimulation assay. Results in **(B–D)** are shown as mean value ± standard error of the mean for 3 independent experiments (n=3). Each symbol represents the data of an individual donor. Statistical significance is indicated as follows: *p < 0.05, **p < 0.01, by Friedman’s test. **(E)** Normalized stacked bar chart to visualize the partition of the different CD8^+^ T cell populations. **(F)** The ControlDC, TriMixDC and TetraMixDC stimulated T cells were added to 624-MEL GFP^+^ cells that were grown in 3D tumor models. The green objective area, representing the viable tumor cells, was measured using the IncuCyte Zoom live cell imaging system. The decline in the green fluorescent area relative to the start value is depicted for TetraMixDC, TriMixDC and ControlDC stimulated T cells conditions. Results are shown as mean value ± standard error of the mean for 2 independent experiments (n=2).

We investigated if TetraMixDCs could prime CD8^+^ T_N_ cells and shift them into activated cytotoxic and memory T cells. CD8^+^ T_N_ cells, defined as CD45RA^+^CD62L^+^CD45RO^-^CD95^-^, were obtained with a purity of approximately 90% (**Figures 7A, B**). These were co-cultured with moDCs that were electroporated with Melan-A/MART-1 (A27L) mRNA and control mRNA (tNGFR), TriMix or TetraMix mRNA at a moDC to T cell ratio of 1 to 10. Three rounds of stimulation at a weekly interval were performed after which the stimulated CD8^+^ T cells were analyzed. We observed that both antigen presenting TriMixDCs and TetraMixDCs showed an increased capacity to activate Melan-A/MART-1 (A27L)-specific CD8^+^ T cells compared to antigen presenting moDCs co-electroporated with control mRNA (**Figure 7C**).

Next we verified the activation status of the CD8^+^ T cells through screening for known T cell activation markers: CD25, CD69, OX40 and CD137. All activation markers were upregulated for T cells stimulated with TriMixDCs and TetraMixDCs. We also observed an up-regulation of the inhibitory checkpoint proteins PD-1 and CTLA-4 in conditions where CD8^+^ T cells were stimulated with TriMixDCs or TetraMixDCs (**Figure 7D**).

Moreover, we evaluated the phenotype of the stimulated T cells by monitoring the following CD8^+^ T cells subsets (48, 49): T_N_ cells (CD45RA^+^CD62L^+^CD45RO^-^CD95^-^), T_SCM_ cells (CD45RA^+^CD45RO^-^CD62L^+^CD95^+^), T_CM_ cells (CD45RA^-^CD45RO^+^CD62L^+^CD95^+^), effector memory T cells (T_EM_; CD45RA^-^CD45RO^+^CD62L^-^CD95^+^) and effector memory re-expressing CD45RA T cells (T_EMRA_; CD45RA^+^CD45RO^-^CD62L^-^CD95^+^). We observe a gradual differentiation from CD8^+^ T_N_ cells to T_SCM_ cells, T_EM_ and T_CM_ cells in the TriMixDC and TetraMixDC conditions (**Figure 7E**).

Finally, we show that the stimulated CD8^+^ T cells were able to kill GFP^+^ 624-MEL cells, in an *in vitro* killing assay. The 624-MEL cell line is HLA-A2^+^ and expresses the Melan-A/MART-1 (A27L) antigen. The 3D tumor models were co-cultured with the stimulated CD8^+^ T cells in the presence of IL-15. We demonstrated that T cells stimulated with TriMixDCs and TetraMixDCs expressing Melan-A/MART-1 (A27L) could kill 624-MEL cells and inhibit tumor growth, which was in contrast to CD8^+^ T cells stimulated with moDCs co-electroporated with MelanA/MART-1 (A27L) mRNA and control mRNA (**Figure 7F**).

## Discussion

Vaccines based on moDCs have been used over the past years to treat many cancer types, with promising but modest results (50). Several obstacles might be responsible for the modest clinical outcome of currently used DC-vaccines. One reason for these poor results might be due to the functional limitation of *in vitro* generated moDCs. Other reasons might be the choice of the target antigens (i.e., tumor associated versus tumor specific antigens) and the tumor-mediated immunosuppression (51). The first limitations can be overcome by influencing the moDC’s status at the time of vaccination (11). It has been shown multiple times that the DC-state might affect the immunogenicity and, in turn, the ability to stimulate a robust anti-tumor response (52–55). Therefore, when employing moDCs it is crucial to reprogram the cells into an optimal cancer vaccine.

In this work, we used a mix of four selected mRNA molecules to increase the moDC’s capacity to activate T cells, enhancing the anticancer immune response, in terms of magnitude, breadth and durability. This novel mRNA mix includes mRNA encoding caTLR4, CD40L, IFN-γ and dIL10Rα. This mRNA mix was developed based on the clinically validated maturation mix called TriMix mRNA, which has shown promising results in several clinical studies (44, 56, 57). During these studies, it was corroborated that IL-12 is a hallmark of a good quality DC-vaccine capable of stimulating T cells. Other studies indicated that the production of IL-12 is a prognostic factor and improves clinical outcome (58). We scrutinized whether IL-12 production by moDCs programmed with CD40L and caTLR4 mRNA could be increased by co-delivering mRNA encoding IFN-γ and dIL10Rα.

To validate the novel maturation mix we compared TetraMix mRNA with the already existing TriMix mRNA. We gained a deeper inside into the effect of TetraMix on the DC-state by performing a transcriptomics analysis after mRNA electroporation. A key difference is that TetraMix mRNA induces an antiviral-like response, whereas TriMix mRNA induces a bacterial-like response after electroporation. Both mRNA mixes are thus inducers of a maturation response in the moDCs but elicit a different reaction. Going deeper into the targeted transcriptomics data, it became clear that TetraMix mRNA induced up-regulation of several genes, which are essential for co-stimulation, migration and the production of important cytokines. Out of the 105 differently expressed genes between TetraMixDCs and TriMixDCs the highest upregulated gene is IL-12A, confirming that we succeeded in generating moDCs that produce high IL-12 levels. The maturation observed in the transcriptome analysis induced by TetraMix mRNA was validated on phenotype and cytokine levels. According to all these data, we can conclude that moDCs electroporated with TetraMix mRNA shift cell state from an immature to a mature phenotype.

The capacity of moDCs to elicit an immune response and induce cancer-specific T cells is key to serve as a potent vaccine. Both TriMixDCs and TetraMixDCs could stimulate a high percentage of Melan-A/MART-1 (A27L)-specific T cells out of the total CD8^+^ T cell population with an advantage for TetraMixDCs. Moreover, we investigated the ability of TetraMixDCs to prime CD8^+^ T_N_ cells and whether these primed T cells could induce tumor killing. A shift was observed from CD8^+^ T_N_ cells into T_SCM_, T_EM_ and T_CM_ cells in the conditions stimulated with TriMixDCs and TetraMixDCs. The stimulated T cells were capable of killing 624-MEL tumor cells, showing the promise of controlling tumor growth.

The results presented in this study suggest that TetraMix mRNA, and the derived antiviral maturation program it induces, has a high potential for DC-based immunotherapy applications. As the next step, the TetraMix technology needs to be validated in a clinical setting. We believe that by reprogramming moDCs by electroporating TetraMix mRNA, the functional limitation of *in vitro* generated moDCs might be overcome. Furthermore, combination with other approaches that support the activated T cells in the immunosuppressive tumor microenvironment is needed to reach the full potential of cancer vaccines (59). We postulate that the TetraMix technology paves the way towards a next generation of immune cell therapy, a so-called ‘super DC-vaccine’, capable of overcoming the modest results of early generation DC-vaccines.

## Materials and methods

### Cell line

HLA-A*0201^POS^ 624-MEL were provided by S.L. Topalian (National Cancer Insitute, Baltimore, MD, USA) and were cultured in Roswell Park Memorial Institute (RPMI) 1640 medium (Sigma-Aldrich) supplemented with 10% fetal clone I serum (Thermo Scientific), 2mM L-glutamine, 100 U/ml penicillin, 100 µg/ml streptomycin, 1 mM sodium pyruvate, and nonessential amino acids (Sigma-Aldrich). The 624-MEL cells were transduced to have a high GFP expression as described in Awad et al (60).

### Generation of monocyte-derived dendritic cells

Generation of moDCs was performed according to Good Manufacturing Practice (GMP). On day 0, a leukapheresis was performed on healthy donors at the Hematology unit of the university hospital in Brussels (UZ Brussels, Belgium) using an apheresis device (Spectra Optia® apheresis system, TerumoBCT) to collect the peripheral blood mononuclear cell (PBMC) fraction. The leukapheresis product was further processed at the DC-manufacturing unit of the Laboratory for Molecular and Cellular Therapy at the Vrije Universiteit Brussel (LMCT-VUB, Brussels, Belgium). An elutriation procedure (Elutra® Cell Separation System Elutra, TerumoBCT) was performed to enrich monocytes. These monocytes were cultured in a cell culture bag with DC-Medium (CellGenix®), supplemented with human serum albumin (1%, CAF-DCF) and cytokines: 500IU/mL recombinant IL-4 (CellGenix®) and 1000IU/mL recombinant granulocyte macrophage-colony-stimulating factor (Leukine®, Sanofi). Differentiation from monocyte to moDCs was allowed for 60-72 hours by incubating the cells at 37°C and 5% CO_2_. The differentiated moDCs were harvested and, after count and viability assessment, cryopreserved in 5% dimethyl sulfoxide (DMSO) cryopreservation medium (CryoStor® CS5, Biolife Solutions). Cryovials were immediately transferred into a freezing container (Corning® CoolCell^TM^ Freezing container) and placed at -80°C. After overnight incubation at -80°C, vials were stored in the vapor phase of a liquid nitrogen container. From the same leukapheresis material, we stored the monocyte-depleted PBMC fraction as previously described (60).

### Generation of DNA templates for *in vitro* mRNA transcription

Plasmid DNA was generated using the in-house developed plasmid pLMCT (61). gBlocks for the different inserts were purchased from IDT and cloned into pLMCT using the Gibson assembly kit™ (NEB) and XL2-Blue Ultracompetent Cells (Agilent). Cloned plasmids were sequence-verified (Eurofins Genomics) and selected clones were further amplified by MIDI DNA-preparation using QIAGEN Plasmid Kits – (QIAGEN). Each plasmid was linearized overnight by restriction enzyme digestion with BfuAI (NEB) to enable *in vitro* mRNA transcription. A thorough quality control was performed assessing the yield (absorbance at 260/280 nm), integrity (BioAnalyzer 2100, DNA 7500 chip) and sequence (Eurofins Genomics) of each plasmid.

### mRNA synthesis

The *in vitro* transcription (*i*VT) reaction was performed starting from a dsDNA template using a T7 enzyme mix containing: T7 RNA polymerase (Thermo Fisher Scientific), RNase inhibitor (Promega) and inorganic pyrophosphatase (Thermo Fisher Scientific). The reaction buffer mix included 10 mM Clean CAP AG reagent (TriLink Biotech) and 10 mM of each dNTP (adenosine-, guanosine-, cytidine- and uridine-triphosphate, Promega). The reaction was incubated at 37 °C for 2 hours. After incubation, DNaseI exonuclease (Thermo Fisher Scientific) was added to the reaction mix and incubated for 15 minutes at 37 °C for the removal of residual dsDNA template. Enzymatic activity was stopped by adding 1.5 volumes of 40 mM EDTA solution to the reaction mix. The mRNA was purified by LiCl-mediated precipitation. Half the reaction volume of 8 M LiCl (Sigma-Aldrich) was added to the mRNA solution and stored at -20 °C overnight. The mRNA sample was centrifugated (15 minutes at 12100 x g) and the obtained pellet was washed with 70% ethanol (Sigma-Aldrich) and subsequently dissolved in RNase-free water (Gibco). A second purification step was performed by NaCl/EtOH precipitation, adding 5M NaCl (Sigma-Aldrich) and absolute ethanol (Sigma-Aldrich). The mRNA was centrifugated for 15 minutes at 14.000 rpm and the obtained pellet was washed with 70% ethanol solution and dissolved in RNase-free water (Gibco). A thorough quality control was performed on the resulting mRNA, including spectrophotometric analysis of optical density for the yield determination and purity (absorbance ratio at 260/280 nm), integrity (BioAnalyzer 2100, RNA 6000 chip) and RNA/cDNA sequence verification after reverse transcription (cDNA kit, NEB & Eurofins Genomics).

### Transfection of mRNA to cells by electroporation

Transfection of mRNA to moDCs was performed by electroporation as described in de Mey et al (61). In short cells were extensively washed in serum-free OptiMEM (Life Technologies, Belgium). The electroporation was performed in 200 µL OptiMEM medium in a 4-mm electroporation cuvette (Cell Projects) using the following parameters: square wave pulse, 500V, 2 ms, 1 pulse using the Gene Pulser Xcell^TM^ device (Biorad, Belgium). Electroporation of moDCs was performed with a total concentration of 100 μg/ml mRNA.

### Transcriptome analysis

Total RNA was extracted from DCs using the RNeasy plus mini kit (Qiagen, 74134) according to manufacturers’ guidelines. A Qubit 4 Fluorometer and the Qubit RNA HS Assay Kit (Thermo Fisher Scientific) were used to assess the RNA yield (on average 81.93 ng/µL ranging from 53 to 132 ng/µL). Absorbance at 260 and 280nm was evaluated using a NanoPhotometer Classic (Implen) with an average A260/A280 value of 2.02 (ranging from 1.942 to 2.055). Samples were run on an Agilent 2100 bioanalyzer using the RNA 6000 nano (5067-1511/1512/1529) kit and the eukaryotic total RNA program. The bioanalyzer electropherograms were analyzed using Agilent 2100 Expert Software to determine the RNA size distribution, the RNA integrity number (RIN) value (on average 9.14, ranging from 8.2-9.6) and the DV200 values (percentage of RNA fragments with a length >200 nucleotides) with an average value of 98.2 (ranging from 91 to 100). RNA (100ng) was analyzed to evaluate gene expression variation using the nanoString nCounter^®^ technology. Samples were hybridized according to manufacturers’ recommendations using the nCounter^®^ Human PanCancer Immune Profiling Panel. Absolute counts were quantified by the nCounter digital analyzer (nCounter MAX Analysis System). Counts were normalized using the RUVSeq method adjusted for nanoString nCounter^®^ gene expression analysis as described by Bhattacharya et al (62). Further data analysis was performed in R. Differential gene expression analysis was done using the DESeq2 package. Principal Component Analysis (PCA) was performed using variance-stabilized counts. Genes were marked as differentially expressed when adjusted p-value < 0.1 and log2 fold change threshold was set above or below 1 and -1, respectively. Gene ontology analysis for biological processes, was calculated using the clusterProfiler package. Biological processes with an adjusted p-value below 0.25 were considered as statistically significant. Gene pattern analysis was performed using the DEGreport package. Plots were generated using the ggplot2, EnhancedVolcano and pheatmap packages in R.

### T-cell stimulation

CD8^+^ T cells were isolated from monocyte-depleted PBMCs by magnetically-activated cell sorting (MACS) using positive selection with human anti-CD8 microbeads according to the manufacturer’s instructions (Miltenyi Biotec). When indicated CD8^+^ T_N_ cells were isolated from monocyte-depleted PBMCs by MACS using the CD8^+^ T-cell isolation kit, anti-CD45RO and anti-CD57 microbeads (Miltenyi Biotec). In that case, the monocyte-depleted PBMCs were depleted from CD45RO and CD57 positive cells, after which a positive selection was performed for CD8^+^ T cells. The T cells were co-cultured with moDCs that were electroporated with mRNA encoding GFP, tNGFR, TriMix, TetraMix or Melan-A/MART-1 following the protocol described by Bonehill et al (53). Evaluation of the CD8^+^ T-cell specificity for Melan-A/MART-1 was assessed at different time points using flow cytometry. When indicated the CD8^+^ T cells were phenotyped by flow cytometry or used for the *in vitro* killing assay.

### Flow cytometry

Cells were harvested and washed twice with phosphate buffered saline containing 1% bovine serum albumin (flow cytometry buffer). The following antibody cocktail was used to phenotype moDCs: anti-CD11c-AF700 (clone BLY6, Becton Dickinson), anti-HLA-DR-PE-Cy7 (clone G466, Becton Dickinson), anti-HLA-ABC-APC-Cy7 (clone W6/32, Becton Dickinson), anti-CD40-PE (clone 5C3, Biolegend), anti-CD80-BV605 (clone L307.4 Becton Dickinson), anti-CD83-BV421 (clone HB15e, Biolgend), anti-CD86-FITC (clone FUN-1, Becton Dickinson), anti-CCR7-APC (clone G043H7, Biolegend) and anti-PD-L1-PE-CF594 (clone MIH1, Becton Dickinson). T-cell dextramer staining: anti-CD8-BV421 (clone RPA-T8, Biolegend) and MHC dextramer Mel-A-PE and -APC (HLA-A*0201/ELAGIGILTV, WB2162, Immudex). T-cell phenotyping was performed using the following antibodies: anti-CD45RA-BV421 (clone HI100, Biolegend), anti-CD45RO-PerCP-Cy5.5 (clone UCHL1, Biolegend), anti-CD95-P3-CF594 (clone DX2, Biolegend), anti-CCR7-PE (clone G043H7, Biolegend), anti-CD62L-FITC (clone DREG-56, Biolegend) and anti-IL-2Rβ-APC (clone TU27, Biolegend). The antibody cocktail used for the T-cell activation panel was: anti-CD25-AF488 (clone BC96, Biolegend), anti-CD69-PerCP-Cy5.5 (clone FN50, Biolegend), anti-CD134-APC (clone Ber-ACT34, Biolegend), anti-CD137-PE (clone 4B4-1, Biolegend), anti-PD-1-BV421 (clone EH12.2H7, Biolegend) and CTLA-4-PE-Cy7 (clone L3D10, Biolegend). All cells were also stained with Fixable viability dye eflour506 (eBioScience) to discriminate live from dead cells. The whole staining procedure was performed on ice. Data were acquired on the LSR Fortessa flow cytometer and analyzed with Flowjo Software, version 10.0.

### Enzyme-linked immunosorbent assay

IL-12p70 and IL-10 were quantified using the human IL-12p70 (MS238) and human IL-10 (BMS215-2) sandwich ELISA from Thermo Fisher Scientific according to the manufacturer’s instructions.

### *In vitro* 3D melanoma killing assay

The 3D culture melanoma killing assay was performed as described by Awad et al (60). In brief GFP^+^ 624-MEL cells were cultured in ultra-low attachment plates (Greiner Bio-One) in 50 µL of DMEM. After 48h stimulated CD8^+^ T cells were added to the 3D tumor models in RPMI containing 100 ng/ml IL-15 (PeproTech, USA). Green fluorescence confluence of 3D tumors was monitored using the Incucyte® ZOOM live cell imaging system (EssenBio, UK). Each 3D tumor confluence was normalized to the 3D tumor confluence at 0h.

### Statistical analysis

All statistical analyses were performed using GraphPad Prism software v8.4.3. Statistical analysis for all figures was performed by Friedman’s test on ranks with uncorrected Dunn’s test. Statistical significance is indicated as follows: *p < 0.05, **p < 0.01, ***p < 0.001,****p < 0.0001.

## Data availability statement

The datasets presented in this study can be found in online repositories. The names of the repository/repositories and accession number(s) can be found below: https://www.ncbi.nlm.nih.gov/geo/, GSE222841.

## Author contributions

KB, WDM, HL, and KT obtained financial support for this work; KB, LF, KT, and MV designed the experiments; DA, WDM, KDR, PDS, AE, LF, HL, and MV conducted and analyzed the experiments; KB, WDM, LF, KT, and MV wrote the paper. All authors contributed to the article and approved the submitted version.
