# Supplementary material for: An mRNA mix redirects dendritic cells towards an antiviral program, inducing anticancer cytotoxic stem cell and central memory CD8+ T cells
Source: Front Immunol. 2023 Feb 13;14:1111523. doi: 10.3389/fimmu.2023.1111523 (PMC9969480; doi:10.3389/fimmu.2023.1111523)
Supplement: Supplementary file 1 [file Table_1.docx]

Supplementary Information:

**Table S1: Significant upregulated/downregulated genes between DCs electroporated with GFP mRNA and DCs electroporated with TriMix mRNA.**

| Upregulated genes | Log2 Fold change | Adjusted p-value |
| --- | --- | --- |
| IL2RA | 8,06448447 | 4,82E-40 |
| IL1B | 8,01737097 | 4,18E-32 |
| IL1A | 7,08229209 | 2,41E-28 |
| ITGB3 | 4,82235666 | 5,97E-28 |
| CCL5 | 4,7203477 | 3,37E-25 |
| PTGS2 | 7,09041525 | 4,03E-24 |
| IL10 | 2,86185947 | 3,06E-23 |
| ADORA2A | 3,91939279 | 2,11E-21 |
| IFNG | 7,49182013 | 1,49E-20 |
| CD40 | 2,1480898 | 1,99E-19 |
| EBI3 | 4,08903748 | 2,97E-19 |
| SOCS1 | 2,98537952 | 1,02E-17 |
| SERPINB2 | 5,8182649 | 3,20E-17 |
| CXCL5 | 7,44782643 | 1,46E-16 |
| DPP4 | 2,34306881 | 1,82E-16 |
| IL6 | 6,89507103 | 4,33E-16 |
| IL15RA | 3,45005228 | 5,58E-16 |
| MUC1 | 3,14052032 | 3,03E-15 |
| CD274 | 3,46499496 | 1,48E-14 |
| ICAM1 | 2,08567255 | 8,66E-14 |
| TNFRSF9 | 2,90816338 | 1,43E-13 |
| SLAMF1 | 5,03738533 | 1,92E-13 |
| LAG3 | 2,73897748 | 2,59E-13 |
| IL2RB | 2,07969394 | 2,66E-13 |
| IL8 | 4,46347955 | 3,82E-13 |
| CD200 | 2,43781558 | 6,31E-13 |
| OSM | 2,23329875 | 9,50E-13 |
| RIPK2 | 2,69333616 | 1,07E-12 |
| PRG2 | 2,24987081 | 4,15E-12 |
| IDO1 | 6,33738718 | 7,03E-12 |
| IL7R | 3,45452219 | 1,01E-11 |
| CXCL1 | 6,88762534 | 1,01E-11 |
| CCL4 | 3,55341031 | 1,08E-11 |
| CFB | 3,87316996 | 1,82E-11 |
| CTSL | 2,8496213 | 3,95E-11 |
| CXCL13 | 4,65934462 | 4,79E-11 |
| CCL20 | 6,50952455 | 6,10E-11 |
| DUSP4 | 3,61408455 | 6,14E-11 |
| CCL19 | 3,68156732 | 3,79E-10 |
| IRAK2 | 2,44409156 | 5,16E-10 |
| PRF1 | 1,67587908 | 6,27E-10 |
| CD80 | 2,66123926 | 1,04E-09 |
| CD38 | 3,90601736 | 1,11E-09 |
| NFKBIA | 1,55912573 | 1,44E-09 |
| CXCR2 | 2,68855445 | 1,45E-09 |
| CXCR5 | 2,21863921 | 2,50E-09 |
| TNFRSF8 | 2,59924597 | 3,89E-09 |
| CDKN1A | 2,16740721 | 5,98E-09 |
| CCR7 | 2,40456697 | 6,27E-09 |
| FCER1G | 1,39632235 | 7,50E-09 |
| TNFAIP3 | 1,97494969 | 9,72E-09 |
| KIR_Activating_Subgroup_1 | 7,4113613 | 1,47E-08 |
| CXCL3 | 4,76620451 | 1,58E-08 |
| VEGFA | 3,24746813 | 1,87E-08 |
| IRF4 | 1,65426961 | 2,50E-08 |
| IL19 | 3,96800569 | 2,67E-08 |
| CCR3 | 2,71615712 | 2,78E-08 |
| STAT4 | 3,79720288 | 3,10E-08 |
| CCL3 | 3,56914987 | 6,61E-08 |
| PVR | 2,01620293 | 7,97E-08 |
| LAMP3 | 2,22989559 | 1,11E-07 |
| MAGEA1 | 1,95341754 | 1,42E-07 |
| CCL3L1 | 3,56477283 | 2,05E-07 |
| FCGR2A | 2,08448615 | 2,12E-07 |
| TBX21 | 2,55910091 | 2,29E-07 |
| NFKB2 | 1,49348952 | 2,36E-07 |
| IL3RA | 1,85856657 | 2,36E-07 |
| CCL17 | 2,73193469 | 2,36E-07 |
| FPR2 | 3,88838705 | 2,62E-07 |
| SBNO2 | 1,15295376 | 3,50E-07 |
| CCL8 | 3,82504073 | 3,50E-07 |
| AGK | 1,23030424 | 4,27E-07 |
| ISG20 | 3,27091208 | 4,45E-07 |
| PPBP | 2,87317103 | 7,53E-07 |
| ITGA1 | 1,43168871 | 7,70E-07 |
| CD70 | 1,86489346 | 8,91E-07 |
| TREM1 | 3,89963883 | 1,05E-06 |
| IL23A | 4,08670887 | 1,75E-06 |
| IRF1 | 2,20331588 | 1,90E-06 |
| RRAD | 2,07488785 | 2,51E-06 |
| ICOS | 2,15010625 | 2,66E-06 |
| MEFV | 2,2167936 | 3,23E-06 |
| CCL23 | 1,9444968 | 4,53E-06 |
| C1R | 2,82012781 | 5,81E-06 |
| IL9 | 2,63275319 | 6,16E-06 |
| CXCL6 | 3,89093458 | 1,61E-05 |
| IL1RN | 2,57725804 | 1,90E-05 |
| BATF | 1,26859074 | 2,55E-05 |
| CSF2RB | 1,27832082 | 2,61E-05 |
| IL1RL1 | 1,4388294 | 4,07E-05 |
| PLAUR | 1,83549168 | 4,50E-05 |
| SLAMF7 | 1,84543296 | 4,66E-05 |
| ITK | 1,20384395 | 4,99E-05 |
| CD19 | 1,11713396 | 6,27E-05 |
| CSF3 | 2,25346126 | 9,14E-05 |
| FEZ1 | 1,6816448 | 0,00011862 |
| TLR2 | 1,13552797 | 0,00015511 |
| IL12B | 3,63707666 | 0,00018311 |
| CXCL9 | 3,88414472 | 0,00028071 |
| CTLA4 | 2,2793524 | 0,00028836 |
| TNFRSF1B | 1,8869031 | 0,00035502 |
| IL12RB2 | 2,12781751 | 0,00038722 |
| IL15 | 1,24155385 | 0,00043716 |
| IL18RAP | 1,35179132 | 0,0004758 |
| TAP1 | 1,50924591 | 0,00056594 |
| IL1RAPL2 | 1,25981849 | 0,0006168 |
| NCR1 | 2,33710969 | 0,00075466 |
| NT5E | 3,30222539 | 0,00079445 |
| CXCL2 | 2,83471839 | 0,00084057 |
| RUNX3 | 1,17974617 | 0,00125721 |
| NLRC5 | 1,30449561 | 0,00155345 |
| CDK1 | 1,32552503 | 0,00174491 |
| NLRP3 | 1,481151 | 0,00190182 |
| SH2D1B | 1,26948869 | 0,00215277 |
| JAK3 | 1,94929552 | 0,00230253 |
| VEGFC | 1,97591592 | 0,00234501 |
| TNFSF10 | 1,36394682 | 0,00287846 |
| IL5 | 2,11489363 | 0,00295027 |
| IL18R1 | 1,63672864 | 0,00305541 |
| CSF2 | 2,48401431 | 0,00325844 |
| LTA | 1,97652421 | 0,00336073 |
| CD7 | 1,11657974 | 0,00366521 |
| PBK | 1,26078081 | 0,00564096 |
| IL27 | 1,86745528 | 0,00873086 |
| ETS1 | 1,37838999 | 0,00890449 |
| C1S | 1,91258444 | 0,01000372 |
| ITGA2B | 1,13201469 | 0,01142052 |
| GZMB | 2,43661269 | 0,01240369 |
| CXCR6 | 1,00488596 | 0,0130227 |
| IL21 | 1,19138796 | 0,01353791 |
| TNFSF4 | 1,15840227 | 0,01416322 |
| IFITM1 | 2,35677289 | 0,01424583 |
| CCL14 | 1,4189887 | 0,01705699 |
| CXCL10 | 2,05455924 | 0,0218925 |
| FYN | 1,08504204 | 0,02469784 |
| FCGR2B | 1,78713666 | 0,02815396 |
| LILRA5 | 1,13637845 | 0,04241439 |
| ITGB4 | 1,08685312 | 0,04301298 |
| CCL1 | 1,26221514 | 0,05729339 |
| TNF | 1,31437068 | 0,08402487 |
| IFI27 | 1,2802623 | 0,09494627 |
| S100A12 | 1,20828499 | 0,09701126 |
| Downregulated genes | **Log2 Fold change** | **Adjusted p-value** |
| PECAM1 | -3,7557842 | 1,92E-50 |
| CCR2 | -4,0548347 | 7,90E-42 |
| CLEC7A | -2,6606074 | 1,82E-29 |
| PDGFC | -2,9687311 | 2,25E-29 |
| AMICA1 | -4,1663316 | 6,26E-26 |
| CD4 | -3,1393475 | 4,55E-25 |
| LY86 | -3,1836708 | 1,13E-21 |
| TLR5 | -3,0847306 | 4,54E-21 |
| ITGAM | -2,7199703 | 9,10E-21 |
| CD9 | -5,3920101 | 7,38E-18 |
| CD36 | -4,068154 | 3,20E-17 |
| APOE | -3,4266459 | 1,28E-13 |
| LGALS3 | -2,2182501 | 1,81E-13 |
| IFNGR1 | -1,8284466 | 5,20E-13 |
| FOS | -2,9277338 | 6,23E-13 |
| CCL24 | -6,0510932 | 1,34E-12 |
| A2M | -2,3614771 | 1,45E-12 |
| CASP10 | -2,7850793 | 2,83E-12 |
| CARD9 | -2,4645867 | 8,86E-12 |
| MRC1 | -3,4537125 | 1,66E-11 |
| HLA-DPB1 | -2,7066653 | 2,40E-11 |
| CSF1R | -2,510069 | 3,06E-11 |
| LAMP1 | -1,22351 | 1,45E-10 |
| CD1C | -3,1939074 | 4,23E-10 |
| HLA-DPA1 | -2,3926494 | 5,14E-10 |
| INPP5D | -2,2256219 | 8,26E-10 |
| PYCARD | -2,5131935 | 1,14E-09 |
| ITGB2 | -2,3865804 | 1,44E-09 |
| HLA-DRB4 | -2,3979665 | 1,61E-09 |
| CD74 | -2,040596 | 4,71E-09 |
| TREM2 | -4,3256306 | 5,50E-09 |
| MEF2C | -1,2912628 | 8,92E-09 |
| ITGAX | -1,5780649 | 1,73E-08 |
| CD68 | -1,4840248 | 1,75E-08 |
| SYK | -1,4971233 | 4,48E-08 |
| HLA-DMB | -3,021092 | 6,09E-08 |
| LRP1 | -2,2301858 | 1,12E-07 |
| NFATC3 | -1,4972516 | 1,24E-07 |
| MSR1 | -3,0357532 | 1,75E-07 |
| HLA-DMA | -2,3468568 | 5,69E-07 |
| HLA-DRB3 | -2,2499497 | 7,30E-07 |
| HLA-DRA | -2,074148 | 8,84E-07 |
| CFD | -1,3932488 | 1,23E-06 |
| ICOSLG | -2,1240611 | 2,40E-06 |
| BLNK | -1,3867454 | 2,44E-06 |
| GUSB | -1,3483351 | 3,14E-06 |
| F13A1 | -3,0736832 | 3,53E-06 |
| MAF | -2,5412507 | 6,16E-06 |
| CD81 | -1,2435965 | 6,16E-06 |
| COLEC12 | -2,1853082 | 6,28E-06 |
| TP53 | -1,7101894 | 6,56E-06 |
| PPARG | -1,8023055 | 8,64E-06 |
| CYBB | -2,4102069 | 9,10E-06 |
| G6PD | -1,7969288 | 1,07E-05 |
| SMAD3 | -1,6575696 | 1,35E-05 |
| CD1B | -2,0643502 | 2,15E-05 |
| EGR2 | -2,6091504 | 2,51E-05 |
| NRP1 | -1,1893908 | 2,68E-05 |
| CD84 | -2,0177519 | 2,86E-05 |
| TAB1 | -1,052085 | 5,32E-05 |
| TFEB | -1,334082 | 5,61E-05 |
| CD180 | -2,2371929 | 6,31E-05 |
| IL16 | -1,6799121 | 9,14E-05 |
| LY96 | -1,3539283 | 9,82E-05 |
| CKLF | -1,5951588 | 0,00010159 |
| CCL13 | -1,935557 | 0,00014949 |
| SPN | -2,6174492 | 0,00027615 |
| KLRB1 | -1,5724669 | 0,00027615 |
| FN1 | -3,8072066 | 0,00032201 |
| LY9 | -1,6441613 | 0,00037379 |
| ECSIT | -1,2284308 | 0,00038274 |
| CD37 | -1,0772627 | 0,00038722 |
| MERTK | -3,0284094 | 0,00039858 |
| C3 | -1,1713259 | 0,00044391 |
| MRPS5 | -1,0701924 | 0,00045121 |
| MAGEB2 | -1,5728796 | 0,00056251 |
| NCF4 | -1,0272655 | 0,00062945 |
| AICDA | -1,3575756 | 0,00090015 |
| IL1R2 | -2,4954047 | 0,00099179 |
| PTPRC | -1,2216598 | 0,00108689 |
| BTK | -1,084706 | 0,00117154 |
| IFIT1 | -1,6338345 | 0,00138424 |
| TNFSF8 | -1,9029885 | 0,00238691 |
| ITGA6 | -1,7803547 | 0,00248204 |
| PPIA | -1,5525429 | 0,00261407 |
| TLR7 | -1,446174 | 0,00265798 |
| S100B | -1,7571787 | 0,00287846 |
| TLR3 | -1,3972074 | 0,00295739 |
| CMKLR1 | -1,1835163 | 0,00308284 |
| ICAM3 | -1,1330191 | 0,00331991 |
| CFP | -1,3315136 | 0,00344641 |
| IRF5 | -1,2441855 | 0,00358377 |
| TNFSF14 | -2,1458851 | 0,00431816 |
| C1QA | -1,3744223 | 0,00439129 |
| SF3A3 | -1,0372772 | 0,00488611 |
| CLEC5A | -2,3263244 | 0,00590053 |
| RPS6 | -1,0879926 | 0,00598027 |
| C5 | -1,0583899 | 0,00708577 |
| CD1A | -1,4895465 | 0,01099501 |
| PIK3CD | -1,1780821 | 0,01181333 |
| NCAM1 | -1,1581915 | 0,01424583 |
| TNFRSF11A | -1,7058174 | 0,01734582 |
| HMGB1 | -1,118028 | 0,01819729 |
| LTB | -1,3618401 | 0,01980897 |
| POU2AF1 | -6,008345 | 0,02250631 |
| FCER1A | -1,0504031 | 0,02843384 |
| DUSP6 | -1,1676577 | 0,031503 |
| CSF1 | -1,650794 | 0,03546049 |
| ENG | -1,0405361 | 0,04157202 |
| TNFSF13 | -1,0553999 | 0,05941507 |
| CXCL12 | -1,3579329 | 0,06492902 |
| EGR1 | -1,1617701 | 0,06609742 |
| CD22 | -1,1619286 | 0,07036655 |

**Table S2: Significant upregulated/downregulated genes between DCs electroporated with GFP mRNA and DCs electroporated with TetraMix mRNA.**

| Upregulated genes | Log2 Fold change | Adjusted p-value |
| --- | --- | --- |
| CD40 | 3,49273611 | 1,31E-50 |
| IL2RA | 8,88504369 | 5,96E-49 |
| CCL19 | 7,82524623 | 3,06E-43 |
| CCL5 | 5,95892957 | 3,75E-40 |
| IL1A | 7,80608155 | 1,09E-34 |
| IL10 | 3,4707938 | 3,02E-34 |
| EBI3 | 5,41972808 | 1,17E-33 |
| IL19 | 7,93236 | 8,24E-31 |
| IL12A | 6,84603387 | 9,16E-31 |
| IL1B | 7,74026418 | 1,61E-30 |
| CD59 | 1,90874984 | 4,23E-30 |
| SOCS1 | 3,89342285 | 4,77E-30 |
| CCR7 | 4,52262969 | 1,16E-29 |
| ADORA2A | 4,58724917 | 1,62E-29 |
| IL23A | 9,13229942 | 5,32E-29 |
| CD70 | 3,85080749 | 3,45E-27 |
| OSM | 3,20644128 | 4,08E-26 |
| IFNG | 8,36367063 | 7,55E-26 |
| IL6 | 8,73877351 | 7,99E-26 |
| CD80 | 4,41095548 | 1,42E-25 |
| IL15RA | 4,35442862 | 1,87E-25 |
| IL12B | 9,37236674 | 1,01E-24 |
| ITGB3 | 4,43507742 | 3,51E-24 |
| LAG3 | 3,68630091 | 3,54E-24 |
| ITGA1 | 2,71121929 | 3,87E-23 |
| IRF4 | 2,81134002 | 8,44E-23 |
| TNFRSF9 | 3,73143801 | 2,10E-22 |
| IRAK2 | 3,69241239 | 3,77E-22 |
| CD200 | 3,18309064 | 3,99E-22 |
| PTGS2 | 6,68736791 | 5,08E-22 |
| LAMP3 | 3,80960638 | 5,32E-21 |
| CSF2 | 6,93191293 | 3,78E-19 |
| CCL1 | 5,15602049 | 4,50E-19 |
| ISG20 | 5,42178552 | 4,76E-18 |
| SERPINB2 | 5,83490445 | 1,05E-17 |
| DPP4 | 2,32802195 | 1,32E-16 |
| SLAMF1 | 5,51326321 | 2,56E-16 |
| CD274 | 3,63067663 | 2,71E-16 |
| CCL4 | 4,19861348 | 2,82E-16 |
| CD38 | 5,10514274 | 3,30E-16 |
| RUNX3 | 2,7681702 | 4,27E-16 |
| AGK | 1,90571044 | 4,44E-16 |
| ICAM1 | 2,17364343 | 2,52E-15 |
| LTA | 4,90393286 | 2,71E-15 |
| IL8 | 4,71110173 | 6,61E-15 |
| IDO1 | 7,06700633 | 6,87E-15 |
| IL27 | 4,99493392 | 1,27E-14 |
| PRG2 | 2,45232866 | 1,39E-14 |
| IL3RA | 2,66304365 | 2,24E-14 |
| TNFAIP3 | 2,56209041 | 2,43E-14 |
| TNFRSF8 | 3,23318478 | 5,47E-14 |
| MEFV | 3,40026606 | 1,22E-13 |
| CCL3 | 4,76776658 | 1,29E-13 |
| CFB | 4,1850203 | 1,60E-13 |
| MUC1 | 2,90594091 | 1,82E-13 |
| CCL3L1 | 4,89750018 | 2,05E-13 |
| CDKN1A | 2,66440175 | 2,53E-13 |
| PRF1 | 1,93075703 | 3,13E-13 |
| IFIT2 | 2,82875485 | 6,82E-13 |
| NFKBIA | 1,80003207 | 1,03E-12 |
| RRAD | 2,94333585 | 2,07E-12 |
| CCL20 | 6,82741111 | 3,18E-12 |
| NFKB2 | 1,94385479 | 4,93E-12 |
| ISG15 | 3,63786904 | 6,75E-12 |
| IL2RB | 1,92756282 | 8,21E-12 |
| TNFSF10 | 2,90128687 | 9,04E-12 |
| PVR | 2,47541419 | 1,41E-11 |
| ICOS | 2,96753772 | 1,48E-11 |
| IL15 | 2,20962735 | 2,76E-11 |
| CXCR5 | 2,41797884 | 3,34E-11 |
| TBX21 | 3,13239752 | 8,06E-11 |
| RIPK2 | 2,39739608 | 1,58E-10 |
| CXCL9 | 6,40493056 | 3,05E-10 |
| CXCL1 | 6,24098442 | 4,84E-10 |
| CXCL13 | 4,26358782 | 1,29E-09 |
| IL7R | 3,02481569 | 1,89E-09 |
| DUSP4 | 3,2800369 | 2,17E-09 |
| STAT4 | 4,02708774 | 2,40E-09 |
| DDX58 | 1,64805381 | 5,50E-09 |
| CSF3 | 3,19370417 | 6,67E-09 |
| SLAMF7 | 2,53364488 | 6,72E-09 |
| IFI27 | 3,88976507 | 9,20E-09 |
| CXCL10 | 4,7185442 | 9,47E-09 |
| CXCR2 | 2,50460312 | 1,37E-08 |
| CCR3 | 2,72021671 | 1,71E-08 |
| TARP | 3,03366258 | 1,89E-08 |
| IRF1 | 2,53735827 | 1,98E-08 |
| TREM1 | 4,38040941 | 2,17E-08 |
| TNF | 3,75279288 | 2,95E-08 |
| CCL17 | 2,84665868 | 4,42E-08 |
| FCGR2A | 2,11940773 | 8,58E-08 |
| CXCL11 | 4,50686471 | 8,83E-08 |
| KIR_Activating_Subgroup_1 | 6,93768112 | 9,39E-08 |
| NLRC5 | 2,0601096 | 1,14E-07 |
| VEGFC | 3,20703697 | 1,27E-07 |
| IL1RN | 3,06598581 | 1,71E-07 |
| IL1RL1 | 1,75341877 | 1,99E-07 |
| FEZ1 | 2,15149261 | 3,05E-07 |
| IL5 | 3,37573795 | 3,55E-07 |
| IFIH1 | 1,69201363 | 5,84E-07 |
| IFITM2 | 1,63913662 | 6,35E-07 |
| IFITM1 | 4,4415044 | 6,96E-07 |
| IRF7 | 2,02578351 | 8,51E-07 |
| CXCL5 | 4,45810296 | 9,56E-07 |
| CXCL6 | 4,22716166 | 1,69E-06 |
| CREB5 | 1,36691469 | 2,02E-06 |
| CD19 | 1,2711611 | 2,83E-06 |
| IFNL1 | 3,55142078 | 2,85E-06 |
| CCL8 | 3,46967121 | 3,22E-06 |
| LILRB2 | 1,70953316 | 3,82E-06 |
| NOD2 | 1,6198519 | 4,28E-06 |
| ITK | 1,29644103 | 7,70E-06 |
| HLA-DOB | 1,81577597 | 7,70E-06 |
| IL1RAPL2 | 1,54756837 | 1,09E-05 |
| TAP1 | 1,85845467 | 1,10E-05 |
| FCER1G | 1,04068445 | 1,73E-05 |
| IL12RB2 | 2,47874836 | 1,86E-05 |
| CD83 | 1,88389209 | 1,90E-05 |
| CTSL | 1,82447438 | 2,80E-05 |
| MAGEA1 | 1,57552885 | 3,10E-05 |
| CTLA4 | 2,53092222 | 3,28E-05 |
| NFATC2 | 1,01544762 | 5,01E-05 |
| NCR1 | 2,69732463 | 5,87E-05 |
| IL18RAP | 1,50377675 | 5,99E-05 |
| BATF | 1,18988527 | 6,33E-05 |
| TAP2 | 1,39996421 | 8,09E-05 |
| IL9 | 2,28850684 | 8,40E-05 |
| IL18R1 | 2,05886788 | 9,33E-05 |
| IL10RA | 1,02391974 | 1,16E-04 |
| CCL22 | 1,42000151 | 1,21E-04 |
| IFNB1 | 2,15182474 | 1,67E-04 |
| THBD | 2,15871505 | 1,75E-04 |
| CD2 | 1,08814079 | 2,03E-04 |
| CXCL3 | 3,1029126 | 2,72E-04 |
| IL26 | 1,33477833 | 3,11E-04 |
| PPBP | 2,10744213 | 3,15E-04 |
| CXCR3 | 1,08984084 | 3,16E-04 |
| MX1 | 1,85116475 | 3,28E-04 |
| CSF2RB | 1,07623426 | 3,68E-04 |
| NOS2A | 2,52819821 | 4,20E-04 |
| C1R | 2,15125424 | 5,69E-04 |
| VEGFA | 1,89925812 | 1,29E-03 |
| LILRA4 | 1,2529044 | 1,31E-03 |
| ITGB4 | 1,59136376 | 1,42E-03 |
| ITGA2 | 1,54134791 | 1,45E-03 |
| PLAUR | 1,39037206 | 2,04E-03 |
| IL13 | 1,25875611 | 2,10E-03 |
| IFNA1 | 1,41870233 | 2,13E-03 |
| IL32 | 1,64347356 | 2,47E-03 |
| IFNL2 | 2,54184789 | 2,64E-03 |
| IL22 | 2,02634754 | 2,65E-03 |
| CXCL2 | 2,45566101 | 3,48E-03 |
| NT5E | 2,82036262 | 3,85E-03 |
| CCL14 | 1,62915555 | 4,36E-03 |
| OAS3 | 1,08236314 | 4,78E-03 |
| GZMK | 1,23795814 | 5,80E-03 |
| CD7 | 1,02287644 | 7,09E-03 |
| SERPING1 | 2,43147112 | 8,05E-03 |
| GZMB | 2,5039254 | 8,07E-03 |
| CDH1 | 1,11945482 | 8,55E-03 |
| TNFSF4 | 1,18213028 | 9,85E-03 |
| IL21 | 1,20531491 | 1,02E-02 |
| BIRC5 | 2,5510581 | 1,03E-02 |
| ETS1 | 1,29949029 | 1,14E-02 |
| C1S | 1,81860143 | 1,19E-02 |
| ITGA2B | 1,09571301 | 1,24E-02 |
| TICAM1 | 1,1139865 | 1,45E-02 |
| IFNA2 | 1,14723391 | 1,68E-02 |
| JAK3 | 1,47417995 | 2,02E-02 |
| BAGE | 2,45436271 | 2,48E-02 |
| IFIT1 | 1,1419159 | 2,51E-02 |
| VCAM1 | 1,39846457 | 3,39E-02 |
| FPR2 | 1,58604154 | 4,63E-02 |
| LILRA5 | 1,07173773 | 4,92E-02 |
| TNFRSF1B | 1,05581083 | 5,08E-02 |
| CD276 | 1,24733324 | 5,40E-02 |
| LIF | 1,14828148 | 6,11E-02 |
| IL24 | 1,1589267 | 8,43E-02 |
| Downregulated genes | **Log2 Fold change** | **Adjusted p-value** |
| PECAM1 | -4,164305 | 4,57E-61 |
| CLEC7A | -3,7763283 | 6,93E-56 |
| CCR2 | -4,6560118 | 7,61E-51 |
| FOS | -5,3988419 | 2,27E-41 |
| CD4 | -3,9920139 | 1,40E-39 |
| AMICA1 | -5,1453081 | 2,88E-38 |
| ITGB2 | -4,5033592 | 9,11E-32 |
| CD36 | -5,5292838 | 1,34E-30 |
| MRC1 | -5,6644829 | 2,46E-29 |
| BST1 | -3,9135958 | 7,28E-28 |
| LY86 | -3,6656228 | 8,52E-28 |
| PDGFC | -2,8321407 | 1,44E-27 |
| CSF1R | -4,2961428 | 2,29E-27 |
| ITGAM | -3,1008012 | 5,81E-27 |
| LRP1 | -4,1546424 | 1,53E-23 |
| TLR5 | -3,2688802 | 6,80E-23 |
| CD99 | -1,7536361 | 1,47E-22 |
| LY96 | -3,215416 | 2,85E-22 |
| CD68 | -2,4305373 | 1,79E-21 |
| IFNGR1 | -2,3133931 | 1,07E-20 |
| LAMP1 | -1,7006869 | 6,89E-20 |
| HLA-DRB4 | -3,5222798 | 9,54E-20 |
| SYK | -2,3302503 | 2,26E-18 |
| CD9 | -5,3382722 | 6,26E-18 |
| APOE | -3,9399314 | 6,83E-18 |
| HLA-DMA | -3,8272215 | 4,37E-17 |
| IL18 | -2,6308949 | 1,02E-16 |
| HLA-DPB1 | -3,2861767 | 1,39E-16 |
| CYBB | -4,2672682 | 2,94E-16 |
| A2M | -2,6240153 | 1,75E-15 |
| HLA-DMB | -4,3059156 | 2,43E-15 |
| CASP10 | -3,2450348 | 2,73E-15 |
| CD81 | -1,9891833 | 6,44E-14 |
| CD1C | -3,848109 | 1,57E-13 |
| CCR1 | -2,1121274 | 3,52E-13 |
| MEF2C | -1,6007211 | 5,50E-13 |
| CD33 | -2,642431 | 6,57E-13 |
| PYCARD | -2,9177102 | 1,17E-12 |
| CD74 | -2,4102694 | 1,56E-12 |
| EGR2 | -4,2616891 | 1,78E-12 |
| MAPK3 | -1,4296782 | 2,17E-12 |
| CARD9 | -2,4592138 | 5,57E-12 |
| INPP5D | -2,4520448 | 6,34E-12 |
| HLA-DPA1 | -2,5210015 | 2,67E-11 |
| TREM2 | -4,8485552 | 4,34E-11 |
| CD14 | -3,7642041 | 8,83E-11 |
| CR1 | -3,2537325 | 1,07E-10 |
| NRP1 | -1,7099036 | 4,27E-10 |
| NUBP1 | -1,3618557 | 4,52E-10 |
| CCR5 | -1,5270855 | 5,62E-10 |
| G6PD | -2,4203783 | 1,02E-09 |
| HLA-DRB3 | -2,6947698 | 1,29E-09 |
| CKLF | -2,3988423 | 1,29E-09 |
| LGALS3 | -1,813291 | 1,31E-09 |
| MAF | -3,3282868 | 1,57E-09 |
| HLA-DRA | -2,4668021 | 2,22E-09 |
| GUSB | -1,6893178 | 3,13E-09 |
| LCP1 | -1,170929 | 3,52E-09 |
| NCF4 | -1,6944572 | 3,60E-09 |
| ENG | -2,7331823 | 3,72E-09 |
| ZC3H14 | -1,2104406 | 1,60E-08 |
| CFD | -1,6070852 | 1,69E-08 |
| MAPK14 | -1,1687146 | 1,85E-08 |
| TNFRSF1A | -1,1988753 | 6,37E-08 |
| TAB1 | -1,3568034 | 1,04E-07 |
| TLR6 | -1,4090228 | 1,18E-07 |
| MAGEB2 | -2,4796186 | 1,38E-07 |
| CD1B | -2,5794903 | 1,44E-07 |
| PPARG | -2,0907003 | 2,43E-07 |
| IFNAR1 | -1,1268043 | 2,59E-07 |
| NFATC3 | -1,43602 | 2,84E-07 |
| ATF1 | -1,1732734 | 4,43E-07 |
| SMAD2 | -1,018153 | 4,82E-07 |
| MAP3K1 | -1,4475387 | 5,90E-07 |
| C1QA | -2,2687963 | 6,48E-07 |
| HDAC3 | -1,2254456 | 1,16E-06 |
| MERTK | -3,9885612 | 1,53E-06 |
| CLEC4A | -1,7303148 | 1,75E-06 |
| ITGA4 | -1,3085754 | 1,87E-06 |
| CD84 | -2,2457855 | 1,89E-06 |
| HMGB1 | -2,0571079 | 3,24E-06 |
| TP53 | -1,7384419 | 3,30E-06 |
| CD209 | -1,0833251 | 3,79E-06 |
| CCL13 | -2,2874322 | 3,86E-06 |
| IL17RA | -1,4696574 | 4,01E-06 |
| CMKLR1 | -1,7325149 | 5,26E-06 |
| F13A1 | -2,9642718 | 5,86E-06 |
| CCL24 | -3,8193061 | 5,99E-06 |
| TOLLIP | -1,0955 | 6,58E-06 |
| SIGIRR | -1,4264966 | 6,70E-06 |
| TLR8 | -1,5354184 | 1,03E-05 |
| BLNK | -1,2804226 | 1,06E-05 |
| MYD88 | -1,0504076 | 1,16E-05 |
| TNFSF12 | -1,8662747 | 1,60E-05 |
| SMAD3 | -1,6203748 | 1,65E-05 |
| CSF3R | -1,8620269 | 1,81E-05 |
| ABL1 | -1,0415765 | 2,14E-05 |
| PIK3CD | -1,8677951 | 2,35E-05 |
| GPI | -1,4152534 | 2,47E-05 |
| MAPK1 | -1,0002633 | 2,87E-05 |
| CCL2 | -2,5608433 | 3,28E-05 |
| MSR1 | -2,3971388 | 3,28E-05 |
| BTK | -1,3367359 | 3,43E-05 |
| TFEB | -1,320493 | 5,07E-05 |
| CD163 | -2,5285014 | 5,42E-05 |
| CD37 | -1,2015278 | 6,16E-05 |
| IL16 | -1,6585592 | 8,40E-05 |
| NFATC1 | -1,2422545 | 1,01E-04 |
| AICDA | -1,5561081 | 1,33E-04 |
| SF3A3 | -1,3510157 | 1,33E-04 |
| IL1R2 | -2,7637328 | 1,74E-04 |
| IRAK1 | -1,5261982 | 1,79E-04 |
| ITGAX | -1,0401288 | 2,33E-04 |
| MRPS5 | -1,0904863 | 2,56E-04 |
| KLRB1 | -1,5108341 | 3,67E-04 |
| COLEC12 | -1,6680339 | 3,71E-04 |
| FCGR3A | -2,0404987 | 3,81E-04 |
| PTPRC | -1,284063 | 4,16E-04 |
| CD180 | -1,9002494 | 5,97E-04 |
| C5 | -1,3145692 | 7,42E-04 |
| CTSS | -1,0847492 | 8,43E-04 |
| MNX1 | -3,7075806 | 8,68E-04 |
| SPN | -2,3209311 | 1,07E-03 |
| SYT17 | -1,1689853 | 1,15E-03 |
| ANP32B | -1,146551 | 1,22E-03 |
| LILRB3 | -1,1322967 | 1,33E-03 |
| TGFB1 | -1,3386496 | 1,49E-03 |
| NCAM1 | -1,4161399 | 2,15E-03 |
| CCL18 | -1,2297452 | 2,19E-03 |
| LAIR2 | -1,1099061 | 2,46E-03 |
| FCER1A | -1,3862245 | 2,92E-03 |
| ILF3 | -1,0328981 | 2,94E-03 |
| ITGA6 | -1,6791093 | 3,52E-03 |
| RPS6 | -1,1177844 | 3,73E-03 |
| PPIA | -1,4281382 | 4,78E-03 |
| CXCR4 | -1,0239676 | 4,90E-03 |
| SPP1 | -1,3799754 | 5,04E-03 |
| TNFSF14 | -2,0319536 | 5,80E-03 |
| MS4A2 | -1,2361074 | 5,99E-03 |
| TLR7 | -1,2822852 | 6,41E-03 |
| FN1 | -2,8608789 | 6,69E-03 |
| IRF5 | -1,1318644 | 7,03E-03 |
| HLA-DQB1 | -1,5187277 | 7,36E-03 |
| CTSW | -1,047307 | 8,07E-03 |
| LY9 | -1,2134818 | 8,10E-03 |
| TNFSF13 | -1,3961528 | 8,47E-03 |
| LTB | -1,4373645 | 1,12E-02 |
| CD1A | -1,4218079 | 1,26E-02 |
| TLR3 | -1,098942 | 1,66E-02 |
| CMA1 | -1,2146872 | 1,67E-02 |
| TNFRSF10C | -1,1986985 | 1,68E-02 |
| POU2AF1 | -5,9967099 | 1,86E-02 |
| EGR1 | -1,4154511 | 1,88E-02 |
| C9 | -1,0342647 | 2,18E-02 |
| TNFRSF12A | -1,1744717 | 2,35E-02 |
| DUSP6 | -1,143649 | 2,96E-02 |
| IL17RB | -1,3389851 | 3,55E-02 |
| S100B | -1,204343 | 3,82E-02 |
| SLC11A1 | -1,0217871 | 4,05E-02 |
| CXCL12 | -1,4548596 | 4,19E-02 |
| TNFSF8 | -1,2734369 | 4,28E-02 |
| FCGR1A | -1,4549563 | 4,32E-02 |
| CD1D | -1,075647 | 4,56E-02 |
| LILRA1 | -1,3392921 | 5,51E-02 |
| IL2 | -1,0928549 | 7,10E-02 |

**Table S3: Significant upregulated/downregulated genes between DCs electroporated with TriMix mRNA and DCs electroporated with TetraMix mRNA.**

| Upregulated genes | Log2 Fold change | Adjusted p-value |
| --- | --- | --- |
| IL12A | 6,3869854 | 6,73E-26 |
| CD59 | 1,32032285 | 1,00E-13 |
| IFIT2 | 2,90165437 | 3,28E-12 |
| CCL19 | 4,14367891 | 3,57E-12 |
| CREB5 | 2,05025885 | 1,09E-11 |
| CCL1 | 3,89380535 | 1,07E-10 |
| IL12B | 5,73529007 | 5,62E-09 |
| IL23A | 5,04559055 | 7,31E-09 |
| IL19 | 3,96435431 | 2,07E-08 |
| CD70 | 1,98591403 | 4,55E-08 |
| CSF2 | 4,44789862 | 4,88E-08 |
| IFIT1 | 2,77575038 | 5,90E-08 |
| CD40 | 1,34464631 | 1,15E-07 |
| ISG15 | 2,85257009 | 5,82E-07 |
| CCR7 | 2,11806271 | 1,13E-06 |
| IFNL1 | 3,79033338 | 3,26E-06 |
| DDX58 | 1,39866383 | 4,99E-06 |
| IL27 | 3,12747865 | 7,26E-06 |
| ITGA1 | 1,27953058 | 1,07E-05 |
| LTA | 2,92740865 | 1,26E-05 |
| RUNX3 | 1,58842402 | 2,18E-05 |
| THBD | 2,53163666 | 4,20E-05 |
| CXCL11 | 3,53753333 | 0,00013998 |
| TARP | 2,20159578 | 0,00022921 |
| CD80 | 1,74971622 | 0,00023169 |
| BCL2 | 1,22165828 | 0,00023245 |
| IRF4 | 1,1570704 | 0,00031505 |
| MX1 | 2,00089408 | 0,00035777 |
| IFI27 | 2,60950277 | 0,00062645 |
| LAMP3 | 1,57971079 | 0,00066778 |
| TNF | 2,43842221 | 0,00161196 |
| TNFSF10 | 1,53734005 | 0,00168505 |
| OAS3 | 1,27556179 | 0,00260248 |
| ITGA2 | 1,62681454 | 0,00301872 |
| ISG20 | 2,15087345 | 0,00330532 |
| ICOSLG | 1,45735828 | 0,00466502 |
| CXCL10 | 2,66398496 | 0,00565953 |
| IRAK2 | 1,24832084 | 0,00567082 |
| IFNB1 | 1,75587557 | 0,00722281 |
| IFITM2 | 1,00635759 | 0,00925933 |
| EBI3 | 1,3306906 | 0,01187971 |
| IFIH1 | 1,00202341 | 0,01256091 |
| IRF7 | 1,17865048 | 0,01642748 |
| ICAM2 | 1,06415484 | 0,01771527 |
| CCL5 | 1,23858188 | 0,02786708 |
| TICAM1 | 1,16372668 | 0,02834971 |
| CCL24 | 2,23178701 | 0,03512039 |
| MEFV | 1,18347246 | 0,03614808 |
| IFNL2 | 2,1035124 | 0,03645219 |
| CSF1 | 1,80850205 | 0,04362559 |
| CD83 | 1,07600332 | 0,05015792 |
| CXCL9 | 2,52078584 | 0,05032189 |
| IFITM1 | 2,08473151 | 0,06755979 |
| BID | 1,06553442 | 0,0779073 |
| CD276 | 1,37016705 | 0,07975655 |
| CLEC5A | 1,7730909 | 0,08396204 |
| IL6 | 1,84370248 | 0,09265769 |
| Downregulated genes | **Log2 Fold change** | **Adjusted p-value** |
| BST1 | -3,1527587 | 5,08E-17 |
| CD14 | -3,5547857 | 1,05E-08 |
| FOS | -2,4711081 | 1,58E-08 |
| CCL23 | -2,453029 | 2,90E-08 |
| BCL6 | -1,2496624 | 2,90E-08 |
| LY96 | -1,8614877 | 2,57E-07 |
| ITGB2 | -2,1167787 | 4,55E-07 |
| IL18 | -1,7482364 | 4,71E-07 |
| CD163 | -3,3054764 | 4,77E-07 |
| CCL18 | -1,9314129 | 3,85E-06 |
| CD33 | -1,8390491 | 6,06E-06 |
| CR1 | -2,4480331 | 9,10E-06 |
| LAIR2 | -1,642244 | 1,88E-05 |
| CCR5 | -1,1391359 | 2,43E-05 |
| LRP1 | -1,9244566 | 3,67E-05 |
| CLEC7A | -1,1157209 | 4,74E-05 |
| CSF1R | -1,7860738 | 8,97E-05 |
| MRC1 | -2,2107705 | 0,00010113 |
| CCR1 | -1,2180547 | 0,00018695 |
| SPP1 | -1,9230184 | 0,00023271 |
| TLR1 | -1,0492053 | 0,00079744 |
| CCL2 | -2,2137304 | 0,00146319 |
| ENG | -1,6926462 | 0,00147175 |
| CSF3R | -1,5412377 | 0,00166621 |
| SLC11A1 | -1,6385194 | 0,00222168 |
| CYBB | -1,8570613 | 0,00227756 |
| TLR8 | -1,1899829 | 0,00269111 |
| CXCL5 | -2,9897235 | 0,00436187 |
| NLRP3 | -1,4763242 | 0,0049174 |
| HLA-DMA | -1,4803647 | 0,00639967 |
| LILRB3 | -1,0620337 | 0,00868579 |
| FPR2 | -2,3023455 | 0,00870247 |
| FCGR1A | -2,0704055 | 0,00921744 |
| CLEC4A | -1,0964226 | 0,00998476 |
| CD36 | -1,4611299 | 0,01355387 |
| IGF1R | -1,0169677 | 0,01652798 |
| HLA-DRB4 | -1,1243133 | 0,01773814 |
| FCGR3A | -1,5928596 | 0,0180122 |
| EGR2 | -1,6525387 | 0,02800925 |
| FCGR2B | -1,8568581 | 0,04760881 |
| CTSL | -1,0251469 | 0,06227569 |
| C3AR1 | -1,0427243 | 0,06236088 |
| VEGFA | -1,34821 | 0,06456069 |
| HLA-DMB | -1,2848236 | 0,06978205 |
